# Time to surgery is not an oncological risk factor in patients with cholangiocarcinoma undergoing curative-intent liver surgery

**DOI:** 10.1038/s41598-023-50842-6

**Published:** 2024-01-18

**Authors:** Anna Mantas, Dong Liu, Carlos Constantin Otto, Lara Rosaline Heij, Daniel Heise, Philipp Bruners, Sven Arke Lang, Tom Florian Ulmer, Ulf Peter Neumann, Jan Bednarsch

**Affiliations:** 1 Department of Surgery and Transplantation, University Hospital RWTH Aachen, Aachen, Germany; 2 Department of Surgery and Transplantation, University Hospital Essen, Essen, Germany; 3 Institute of Pathology, University Hospital RWTH Aachen, Aachen, Germany; 4 Department of Diagnostic and Interventional Radiology, University Hospital RWTH Aachen, Aachen, Germany; 5 Department of Surgery, Maastricht University Medical Center (MUMC), Maastricht, The Netherlands

**Keywords:** Experimental models of disease, Outcomes research

## Abstract

Surgical resection is the only option to achieve long-term survival in cholangiocellular carcinoma (CCA). Due to limitations of health care systems and unforeseeable events, e.g., the COVID pandemic, the time from diagnosis to surgery (time-to-surgery (TTS)) has gained great interest in malignancies. Thus, we investigated whether TTS is associated with the oncological outcome in patients who underwent surgery for CCA. A cohort of 276 patients undergoing curative-intent surgery for intrahepatic and perihilar CCA excluding individuals with neoadjuvant therapy and perioperative mortality between 2010 and 2021 were eligible for analysis. Patients were grouped according to TTS (≤ 30; 31–60; 61–90; > 90 days) and compared by Kruskal–Wallis-analysis. Survival was compared using Kaplan–Meier analysis and characteristics associated with cancer-specific survival (CSS), recurrence-free survival (RFS) and overall survival (OS) using Cox regressions. The median CSS was 39 months (3-year-CSS = 52%, 5-year-CSS = 42%) and the median RFS 20 months (3-year-CSS = 38%, 5-year-CSS = 33%). In univariable Cox regressions, TTS was not associated with CSS (*p* = 0.971) or RFS (*p* = 0.855), respectively. A grouped analysis with respect to TTS (≤ 30 days, n = 106; 31–60 days, n = 134; 61–90 days, n = 44; > 90 days, n = 29) displayed a median CSS of 38, 33, 51 and 41 months and median RFS of 17, 22, 28 and 20 months (*p* = 0.971 log rank; *p* = 0.520 log rank). No statistical difference regarding oncological risk factors were observed between the groups. This study is the first comprehensive analysis of TTS in CCA patients. Within a representative European cohort, TTS was not associated with earlier tumor recurrence or reduced CCS.

## Introduction

Cholangiocellular carcinoma (CCA) is the second most common primary malignant liver tumor^1^. With respect to their anatomical location within the biliary system, CCAs are usually divided into intrahepatic CCA (iCCA), perihilar CCA (pCCA) or distal CCA (dCCA)^2^. Due to their asymptomatic clinical presentation but also the aggressive nature and diagnostic difficulties, CCAs are often diagnosed at advanced disease stages^3^. As the incidence of CCAs worldwide is increasing and the oncological outcome remains dismal compared to other solid malignancies, novel and improved treatment strategies are of upmost importance^4^. To date, surgical resection including lymphadenectomy remains to be the gold standard for curative-intent treatment in localized CCA^5^. Liver resection for CCA, especially for the perihilar CCA, is associated with significant perioperative complications with some studies reporting up to 15% mortality^6^. Subsequently, these patients require a notable amount of medical resources ranging from extended time in operating theatres to postoperative surveillance in ICU, complication management as well as capacities on normal wards due to the usually long hospitalization^7,8^.

During the last two years, the global health systems have shifted resources to encounter the COVID-19 pandemic. Thus, curative intention surgery in oncological patients was frequently delayed and the corresponding impact on clinical outcome investigated^9^. Reduced overall survival (OS) of patients with different malignant diseases due to delayed time to surgery (TTS) in the scenario of surgical-, systemic- (adjuvant, neoadjuvant) and radiotherapy have been described^10^. However, the role of TTS in the oncological outcome of CCA patients remains to be elucidated. Thus, the aim of this study was to investigate the impact of TTS on short- and long-term outcome in CCA patients.

## Material and methods

### Patients

Between 2010 and 2021, all consecutive patients with localized intrahepatic and perihilar CCA with no signs of systemic disease who were treated with curative-intent liver resection at the University Hospital RWTH Aachen (UH-RWTH) were included in this study. Patients who underwent neoadjuvant chemotherapy or exploratory laparotomy and postoperative mortality were excluded to focus on oncological effects of in-hospital surgical treatment. The study was conducted in accordance with the requirements of the Institutional Review Board of the RWTH-Aachen University (EK 22-343), the current version of the Declaration of Helsinki and the good clinical practice guidelines (ICH-GCP). As such, the Institutional Review Board of the RWTH-Aachen University has approved the protocol (No. EK 22-343). Informed consent was obtained from the patients. All clinical data were collected within an institutional hepatobiliary database and analyzed retrospectively. There was no prioritization for patients who underwent surgical resection. Surgery was conducted as soon as possible in every case.

### Staging and surgical technique

All patients who were referred for surgical treatment of CCA to our institution underwent a detailed preoperative clinical work-up including an oncological staging in accordance with common standards and radical surgery with lymphadenectomy^11,12^. Major (≥ 3 segments) and minor (≤ 2 segments) were defined according to common understandings. All surgical specimens were evaluated by an experienced board-certified staff pathologist und classified according to the 8th edition of the Union for international cancer control (UICC).

### Study definitions

TTS was defined as the date difference between the date of diagnosis and the date of surgery. The date of diagnosis was defined as the date of the first radiologic imaging indicating the presence of CCA, e. g., contrast-enhancend ultrasound, magnetic resonance imaging (MRI) or computed tomography (CT) or the first endoscopic retrograde cholangiopancreatography (ERCP).

### Follow-up

In 2010 to 2017, adjuvant therapy was recommended for patients with high risk factors, such as positive nodal status or R1 resection, and later on to every patient with regard to the BILCAP trial^13^. Each patient received a regular follow-up consisting of clinical examinations, standard laboratory blood tests with tumor markers (CA 19-9) and cross-sectional imaging. Additional imaging and/or biopsy was performed if tumor recurrence was suspected in order to confirm the diagnosis as previously described^11,12^.

### Statistical analysis

The primary aim of this study was to investigate whether TTS has a significant oncological effect on cancer-specific survival (CSS) and recurrence-free survival (RFS) in CCA patients. CSS was defined as the period from the date of surgery to the date of tumor-specific death or the last contact if the patient was alive. Deaths not associated with the tumor, e.g. cardiovascular events, were censored at the time of death. RFS was defined as the time from resection to the date of the first recurrence. Patients without tumor recurrence were censored at the time of death or at the last follow-up. Patients suffering perioperative mortality or receiving neoadjuvant treatment were excluded from the oncological analysis. All included patients were divided into groups with respect to TTS (1–30 d, 31–60 d, 61–90 d and over 91 days). Categorical data was compared using chi-square test (expressed through numbers and percentages). To compare continuous variables, the Kruskal–Wallis test was used. Kaplan–Meier method was applied to generate survival curves which were compared with the log-rank test. Variables associated with CSS and RFS were identified using univariate Cox regression analyses. Multivariable cox regression analyses in a backward selection model was applied to significant parameters only. Median follow up was assessed with the reverse Kaplan–Meier method. The level of significance was set to *p* < 0.05 and *p*-values were given for two-sided testing. All analyses were carried out using SPSS Statistics 24 (IBM Corp., Armonk, NY, USA).

### Ethical standards

All procedures performed in studies involving human participants were in accordance with the ethical standards of the institutional and/or national research committee and with the 1964 Helsinki declaration and its later amendments or comparable ethical standards. The study was conducted at the UH-RWTH in accordance with the requirements of the Institutional Review Board of the RWTH-Aachen University (EK 22-343).

## Results

### Patient cohort

Between 2010 and 2021, a total of 276 patients underwent curative-intent liver resection for CCA at our department and met the above-mentioned inclusion criteria. Of note, 161 individuals underwent exploratory laparotomy during the same time. The final study cohort (n = 276) comprised 146 pCCA and 130 iCCA patients with a mean age of 66 years. Most patients were male (55.4%, 153/276) and classified as ASA (American Society of Anesthesiologists classification) III (53.5%, 147/276) score. Major hepatectomies were necessary in almost every case (82.2%, 227/276) which resulted in a R0 resection rate of 86.5% (239/276). Major complications (≥ Clavien-Dindo IIIb) after surgery were observed in 96 patients (27.2%, 75/276). A total of 36.2% (100/276) of the patients received intraoperative PRBC and 42.8% (118/276) intraoperative FFP transfusions. The average hospital stay was 23 days. Further demographic and clinico-pathological details of the cohort are outlined in Table 1. As part of our analyses, we also conducted a subanalysis for both tumors separately. The results are displayed in Supplementary Table 1 and Table 2.

**Table 1:** Patients’ characteristics.

	CCA (n = 276)	Time to surgery subanalysis				*p* value
		1–30 days (n = 95)	31–60 days (n = 121)	61–90 days (n = 34)	> 90 days (n = 26)	
Demographics						
Gender, m/f (%)	153 (55.4)/123 (44.6)	46 (48.4)/49 (51.6)	74 (61.2)/47 (38.8)	21 (61.8)/13 (38.2)	12 (46.2)/14 (53.8)	0.175
Age (years)	66 ± 11	64 ± 11	68 ± 10	67 ± 9	68 ± 9	**0.028**
BMI (kg/m^2^)	26 ± 5	26 ± 5	26 ± 5	27 ± 5	27 ± 6	0.863
Portal vein embolization, n (%)	61 (22.1)	9 (9.5)	29 (24)	13 (38.2)	10 (38.5)	**0.001**
ASA, n (%)						0.328
I	9 (3.3)	3 (3.2)	5 (4.1)	1 (2.9)	0	
II	103 (37.5)	43 (45.7)	37 (30.6)	14 (41.2)	9 (34.6)	
III	147 (53.5)	42 (44.7)	72 (59.5)	19 (55.9)	14 (53.8)	
IV	16 (5.8)	6 (6.4)	7 (5.8)	0	3 (11.5)	
V	0	0	0	0	0	
Tumor, n (%)						0.086
pCCA	146 (52.9)	53 (55.8)	67 (55.4)	17 (50)	9 (34.6)	
iCCA	130 (47.1)	42 (44.2)	54 (44.6)	17 (50)	17 (65.4)	
Preoperative chemotherapy	0	0	0	0	0	n.a
Future liver remnant modulation						
Endoscopic stenting	117 (42.4)	40 (42.1)	54 (44.6)	12 (35.3)	11 (42.3)	0.227
Percutaneous transhepatic biliary drainage	35 (12.7)	10 (10.5)	19 (15.7)	6 (17.6)	0	0.139
Exploratory laparotomies during the same study period, n (%)**	161 (n.a.)	64 (n.a.)	46 (n.a.)	23 (n.a.)	28 (n.a.)	n.a
Clinical chemistry (preoperative)						
AST (U/l)	74 ± 190	97 ± 295	70 ± 117	49 ± 29	42 ± 40	0.060
GGT (U/l)	448 ± 505	501 ± 566	446 ± 491	421 ± 484	302 ± 328	0.219
Total bilirubin (mg/dl)	1.7 ± 3.3	2.7 ± 5	1.4 ± 2	0.8 ± 0.7	0.7 ± 0.5	0.072
Hemoglobin (g/dl)	12.6 ± 1.7	12.6 ± 1.7	12.5 ± 1.7	12.9 ± 1.6	12.7 ± 1.7	0.746
Platelet count (/nl)	296 ± 120	325 ± 122	280 ± 126	291 ± 86	270 ± 98	**0.003**
INR	1.14 ± 1.63	1.03 ± 0.12	1.04 ± 0.11	1.87 ± 4.73	1.07 ± 0.29	0.661
Prothrombin time (%)	97 ± 16	98 ± 16	96 ± 15	96 ± 13	96 ± 19	0.617
CRP (mg/l)	25 ± 38	26 ± 36	26 ± 41	29 ± 49	16 ± 14	0.795
Operative data						
Operative time (minutes)	374 ± 121	364 ± 118	379 ± 122	389 ± 126	361 ± 126	0.611
Operative procedure, n (%)						0.567
Atypical	14 (5.1)	3	7	2	2	
Monosegmentectomy	1 (0.4)	0	0	1	0	
Bisegmentectomy	12 (4.3)	4	6	0	2	
Hemihepatectomy	87 (31.5)	38	32	10	7	
Extended hemihepatectomy^#^	87 (31.5)	31	38	9	9	
Trisectionectomy	43 (15.6)	9	24	7	3	
Hepatoduodenoectomy^†^	8 (2.9)	1	5	1	1	
ALPPS	12 (4.3)	4	4	3	1	
other	12 (4.3)	5	5	1	1	
Laparoscopic resection, n (%)	15 (6.8)	3 (4.2)	8 (8)	1 (3.2)	3 (15)	0.291
Intraoperative PRBC, n (%)	101 (36.6)	40 (42.1)	42 (34.7)	12 (35.3)	7 (26.9)	0.691
Intraoperative FFP, n (%)	120 (43.5)	43 (45.3)	53 (43.8)	16 (47.1)	8 (30.8)	0.824
Intraoperative platelets, n (%)	2 (0.8)	1 (1.1)	0	0	1 (3.8)	0.187
Pathological examination						
R1 resection, n (%)	37 (13.5)	12 (12.8)	14 (11.6)	9 (26.5)	2 (7.7)	0.101
pN category, n (%)						0.365
N0	161 (60.8)	62 (66)	69 (60)	16 (48.5)	14 (60.9)	
N1	104 (39.2)	32 (34)	46 (40)	17 (51.5)	9 (39.1)	
Tumor grading, n (%)						0.878
G1	7 (2.6)	2 (2.2)	3 (2.6)	1 (3)	1 (4.2)	
G2	183 (68.8)	70 (75.3)	75 (64.7)	22 (66.7)	16 (66.7)	
G3	70 (26.3)	21 (22.6)	34 (29.3)	9 (27.2)	6 (25)	
G4	5 (1.9)	0	3 (2.6)	1 (3)	1 (4.2)	
MVI, n (%)	12 (4.4)	4 (4.2)	4 (3.3)	4 (11.8)	0	0.490
LVI, n (%)	55 (21.1)	22 (24.7)	22 (19.5)	9 (26.5)	2 (8)	0.256
pT category n (%)						**0.034**
1	64 (23.1)	21 (22.1)	21 (17.3)	10 (29.4)	12 (46.2)	
2	136 (49.3)	44 (46.3)	67 (55.3)	17 (50.1)	8 (30.7)	
3	54 (19.6)	27 (28.4)	21 (17.4)	2 (5.9)	4 (15.4)	
4	22 (8.0)	3 (3.2)	12 (9.9)	5 (14.7)	2 (7.7)	
Postoperative data						
Intensive care, days	2 ± 7	2 ± 2	3 ± 10	3 ± 6	2 ± 1	0.658
Hospitalization, days	23 ± 25	20 ± 14	25 ± 32	28 ± 25	20 ± 16	0.463
Postoperative complications, n (%)						0.186
No complications	75 (27.2)	28 (29.5)	31 (25.6)	5 (14.7)	11 (42.3)	
Clavien-Dindo I	85 (30.8)	22 (23.2)	45 (37.2)	10 (29.4)	8 (30.8)	
Clavien-Dindo II	116 (60.1)	56 (58.9)	77 (63.6)	22 (64.7)	11 (42.3)	
Clavien-Dindo IIIa	159 (35.9)	30 (31.8)	45 (37.1)	16 (46.9)	8 (30.8)	
Clavien-Dindo IIIb	51 (18.5)	17 (18)	21 (17.3)	9 (26.4)	4 (15.3)	
Clavien-Dindo IVa	19 (6.9)	6 (6.3)	9 (7.4)	4 (11.7)	0	
Clavien-Dindo IVb	5 (1.8)	0	3 (2.5)	2 (5.9)	0	
Clavien-Dindo V	0	0	0	0	0	
Oncologic Data*						
Adjuvant chemotherapy, n (%)	93 (33.8)	39 (41.1)	34 (28.1)	13 (39.4)	7 (26.9)	0.172
Recurrence, n (%)	160 (59)	57 (61.3)	70 (59.3)	21 (61.8)	12 (46.2)	0.554
Median RFS, months (95% CI)	20 (15–25)	17 (9–25)	22 (16–28)	28 (8–48)	20 (0–44)	0.850
Median CSS, months (95% CI)	39 (28–50)	38 (8–68)	33 (21–45)	51 (8–94)	41 (10–72)	0.971
Median OS, months (95% CI)	32 (26–38)	32 (22–42)	31 (25–37)	41 (6–76)	29 (6–52)	0.638

Data presented as mean and standard deviation if not noted otherwise.

*ALPPS* Associating liver partition and portal vein ligation for staged hepatectomy, *ASA* American society of anesthesiologists classification, *AST* aspartate aminotransferase, *BMI* body mass index, *CSS* cancer-specific survival, *EBD* endoscopic biliary drainage, *FFP* fresh frozen plasma, *pCCA* perihilar cholangiocarcinoma, *GGT* gamma glutamyltransferase, *iCCA* intrahepatic cholangiocarcinoma, *INR* international normalized ratio, *LVI* lympho-vascular invasion, *MVI* microvascular invasion, *n./a.* not applicable, *OS* overall survival, *PRBC* packed red blood cells, *RFS* disease free survival, *UICC* Union for international cancer control.

*Data presented as median and interquartile range.

**This data was not included in statistical analysis.

#Right or left hepatectomy were considered to be extended hepatectomies if the middle hepatic vein was removed and the resection was extended into the segments IV or V/VIII, respectively.

^†^Procedures were defined as hepatoduodenoectomy if a major liver resections was combined with the concomitant resection of the pancreatic head.

Significant values are in bold.

Categorized by TTS, 95 patients underwent liver resection within 30 days after diagnosis, 121 patients between 31 and 60 days, 34 between 61 and 90 days and 26 patients after 90 days. Comprehensive group comparisons demonstrated no differences in major demographic and oncological characteristics. However, differences were observed in age (*p* = 0.028), portal vein embolization (*p* = 0.001), preoperative platelet count (0.003) and pT category (0.034). Other parameters showed no statistical differences in distribution. Detailed perioperative results for the 4 subgroups can be found in Table 2.

**Table 2:** Univariable analysis of recurrence-free and cancer-specific survival in cholangiocarcinoma.

	n	Recurrence-free survival (RFS)			Cancer-specific survival (CSS)		
		Median RFS, m (95% CI)	Relative risk (95% CI)/HR	*P* value	Median CSS, m (95% CI)	Relative risk (95% CI)/HR	*P* value
Sex				0.528			0.766
Male	151	23 (14–32)			38 (23–53)		
Female	120	18 (12–24)			41 (24–58)		
Age, years				0.209			0.705
≤ 65	122	17 (12–22)			39 (24–54)		
> 65	149	24 (16–32)			38 (24–52)		
BMI, kg/m^2^				0.427			0.527
≤ 25	126	24 (13–35)			45 (29–62)		
> 25	144	18 (12–24)			33 (18–48)		
PVE				0.641			0.717
No	210	18 (13–23)			38 (28–48)		
Yes	61	27 (10–44)			51 (7–95)		
ASA				0.395			0.182
I/II	110	20 (9–31)			51 (30–73)		
III / IV	160	19 (13–25)			32 (24–40)		
Tumor entity				**0.004**			0.326
pCCA	146	29 (19–39)	1		49 (32–66)		
iCCA	130	13 (9–17)	1.569 (1.149–2.142)		32 (23–41)		
Time to surgery				0.855			0.971
< 30	93	17 (9–25)			38 (8–68)		
30–60	118	22 (16–28)			33 (21–45)		
60–90	34	28 (8–48)			51 (8–94)		
> 90	26	20 (0–44)			41 (10–72)		
AST, U/l				0.241			0.179
≤ 40	135	20 (13–28)			41 (19–63)		
> 40	134	17 (7–27)			33 (16–50)		
GGT, U/l				0.196			0.054
≤ 100	106	20 (9–31)			50 (23–77)		
> 100	156	21 (15–27)			32 (22–42)		
Bilirubin, mg/dl				0.051			0.646
≤ 1	174	19 (13–25)			38 (23–53)		
> 1	96	23 (9–37)			41 (23–59)		
Platelet count, 1/nl				**0.002**			0.806
≤ 250	109	22 (16–28)	1		38 (25–51)		
> 250	161	20 (12–28)	0.993 (0.723–1.363)		41 (22–60)		
INR				**0.040**			**0.002**
≤ 1	115	25 (15–35)	1		61 (39–83)	1	
> 1	153	16 (9–23)	1.396 (1.014–1.923)		31 (26–36)	1.762 (1.234–2.515)	
Hemoglobin, g/dl				**0.002**			**0.002**
≤ 12	92	14 (10–18)	1		27 (21–33)	1	
> 12	178	28 (17–39)	0.602 (0.436–0.831)		51 (32–70)	0.574 (0.405–0.815)	
CRP, mg/l				0.159			**0.010**
≤ 10	127	24 (15–33)			46 (20–72)	1	
> 10	132	18 (11–26)			31 (18–44)	1.578 (1.114–2.236)	
Operative time, min				0.174			0.068
≤ 360	133	23 (13–33)			46 (21–72)		
> 360	138	20 (14–27)			33 (23–43)		
Operative procedure				**0.005**			**0.049**
Monosegmentectomy + bisegmentectomy	27		1			1	
Hemihepatectomy	86		2.586 (1.226–5.456)			2.110 (0.944–4.714)	
Ext. hemihepetectomy + trisectionectomy	143		2.560 (1.241–5.283)			2.268 (1.046–4.914)	
others	15		4.737 (1.953–11.491)			3.636 (1.427–9.261)	
In-hospital PRBC (qualitatively)				**0.002**			**0.001**
No	171	25 (17–33)	1		76 (39–113)	1	
Yes	100	12 (7–17)	1.65 (1.2–2.265)		28 (18–38)	2.014 (1.438–2.821)	
In-hospital FFP (qualitatively)				**0.001**			**0.001**
No	153	25 (18–32)	1		90 (n.a.)	1	
Yes	118	12 (9–15)	1.663 (1.218–2.271)		28 (21–35)	2.15 (1.532–3.02)	
R1 resection				**0.001**			**0.001**
No	215	23 (17–29)	1		49 (32–66)	1	
Yes	37	10 (7–13)	2.089 (0.918–4.751)		16 (8–24)	2.803 (1.838–4.274)	
MVI				**0.001**			**0.001**
No	173	35 (24–47)	1		63 (40–87)	1	
Yes	89	11 (8–14)	2.214 (1.605–3.055)		23 (17–29)	2.285 (1.626–3.210)	
LVI				**0.001**			**0.001**
No	201	30 (20–40)	1		51 (39–64)	1	
Yes	55	10 (6–14)	2.437 (1.687–3.519)		18 (14–23)	2.905 (1.963–4.3)	
Tumor grading				**0.001**			**0.001**
G1/G2	189	29 (20–38)	1		51 (33–69)	1	
G3/G4	68	12 (7–17)	1.977 (1.389–2.816)		20 (10–31)	2.464 (1.7–3.57)	
pN category				**0.001**			**0.001**
N0	157	35 (20–50)	1		63 (40–86)	1	
N1	103	10 (8–13)	2.324 (1.685–3.205)		20 (13–27)	2.696 (1.911–3.803)	
ICU time, days				0.374			0.085
≤ 1	196	20 (13–27)			46 (34–58)		
> 1	74	18 (9–27)			26 (18–35)		
Hospitalization, days				**0.007**			**0.011**
≤ 14	131	25 (11–40)	1		50 (31–69)	1	
> 14	133	16 (11–21)	1.54 (1.12–2.11)		30 (23–37)	1.542 (1.1–2.16)	
Perioperative complications				0.469			0.**023**
Clavien-Dindo I/II/IIIa	215	21 (14–28)			41 (24–58)	1	
Clavien-Dindo IIIb/IV	56	20 (11–29)			31 (15–47)	1.57 (1.06–2.324)	
Adjuvant therapy				**0.011**			0.516
No	181	27 (16–38)	1		45 (31–59)		
Yes	93	15 (11–19)	1.51 (1.095–2.089)		32 (22–42)		

Various parameters are associated with cancer-specific or recurrence-free survival.

*ASA* American society of anesthesiologists classification, *AST* aspartate aminotransferase, *BMI* body mass index, *CSS* cancer-specific survival, *FFP* fresh frozen plasma, *pCCA* perihilar cholangiocarcinoma, *GGT* gamma glutamyltransferase, *iCCA* intrahepatic cholangiocarcinoma, *INR* international normalized ratio, *LVI* lympho-vascular invasion, *MVI* microvascular invasion, *PRBC* packed red blood cells, *PVE* portal vein ligation, *RFS* recurrence-free survival, *UICC* Union for international cancer control.

Significant values are in bold.

When analyzed separately for tumor entities, in patients with pCCA, 53 patients underwent surgery within 30 days after diagnosis, 67 patients between 31 and 60 days, 17 between 61 and 90 days and 9 patients after 90 days. Group comparisons in patients with pCCA showed no major differences except for PVE (*p* = 0.001), total bilirubin (*p* = 0.026), platelet count (*p* = 0.044), and intraoperative platelets (*p* = 0.002). In the case of iCCA, 42 patients with iCCA received surgical treatment within 30 days after diagnosis, 54 patients between 31 and 60 days, 17 patients between 61 and 90 days and 17 patients after 90 days. Concerning group comparisons in patients with iCCA, differences were observed in age (*p* = 0.031), tumor grading (*p* = 0.041) and adjuvant chemotherapy (*p* = 0.068). Detailed perioperative results can be found in Supplementary Table 1 and Table 2.

While not the primary aim of our analysis, patients undergoing exploratory laparotomy without curative surgery were numerically analyzed regarding their TTS. During the study period, a total of 161 patients were surgically explored without an obvious tendency for longer TTS in these patients (TTS 1–30 d: 64; TTS 31–60 d: 46; TTS 61–90 d: 23; TTS > 90 d: 28). Note that these cases were not used for any other statistical analysis and not further interpreted.

Variations in TTS during study period were further investigated. Here, we observed different TTS values in a grouped comparison using two-year-periods (2010/2011: n = 24, median TTS = 48 d; 2012/2013: n = 38, median TTS = 38 d; 2014/2015: n = 48, median TTS = 28 d; 2016/2017: n = 45, median TTS = 36 d; 2018/2019: n = 53, median TTS = 42 d; 2020/2021: n = 68, median TTS = 47 d; *p* < 0.001).

For sensitivity reasons, patients’ characteristics were analyzed including those patients who showed perioperative mortality. To conclude, there were no differences in comparison to the analyses of the cohort without mortality observed. Also, TTS had no effect on CCS (*p* = 0.515), OS (*p* = 0.280) and RFS (*p* = 0.838) in this grouped analysis. Details can be found in Supplementary Table 7.

### Survival analysis

**Figure 1:**
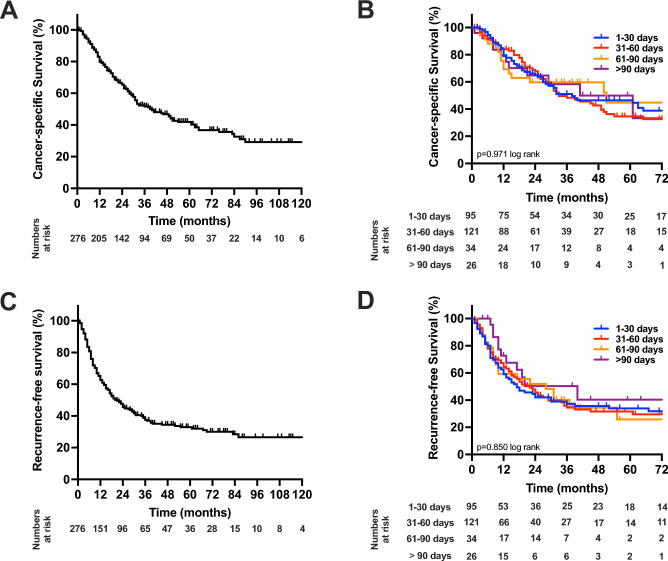
Oncological survival in cholangiocarcinoma stratified by time to surgery. (**A**) Cancer-specific in cholangiocarcinoma. The median CCS was 39 months. (**B**) Cancer-specific in cholangiocarcinoma stratified by time to surgery. The median CSS were 38 months (TTS 1–30 d), 33 months (TTS 31–60 d), 51 months (TTS 61–90 d) and 41 months (TTS > 90 d). (**C**) Recurrence-free survival in cholangiocarcinoma. The median RFS 20 months. (**D**) Recurrence-free survival in cholangiocarcinoma stratified by time to surgery. The median RFS were 17 months, 22 months (TTS 31–60 d), 28 months (TTS 61–90 d) and 20 months (TTS > 90 d). *CCS* cancer-specific survival, *RFS* recurrence-free survival.

After a median follow-up of 52 months, the median CSS of the whole cohort was 39 months (95% confidence interval (CI): 28–50), (3-year-CSS = 52%, 5-year-CSS = 42%), the median RFS 20 months (95% CI 15–25), (3-year-CSS = 38%, 5-year-CSS = 33%), Fig. 1A and C). The median OS of the whole cohort was 32 months (95% CI 26–38). Regarding TTS, the median CSS were 38 months (95% CI 8–68; TTS 1–30 d), 33 months (95% CI 21–45; TTS 31–60 d), 51 months (95% CI 8–94, TTS 61–90 d) and 41 months (95% CI 10–72; TTS > 90 d) without statistical significance (*p* = 0.971 log rank, Fig. 1B). Moreover, the median RFS were 17 months (95% CI 9–25; TTS 1–30 d), 22 months (95% CI 16–28; TTS 31–60 d), 28 months (95% CI 8–48, TTS 61–90 d) and 20 months (95% CI 0–44; TTS > 90 d) without statistical significance (*p* = 0.850 log rank, Fig. 1D). The median OS were 32 months (95% CI 22–42, TTS 1–30 d), 31 months (95% CI 25–37 months; TTS 31–60 d), 41 months (95% CI 6–76; TTS 61–90 d) and 29 months (95% CI 6–52 months; TTS > 90 d) without statistical significance (*p* = 0.638 log rank).

### Survival analysis with respect to tumor type

**Figure 2:**
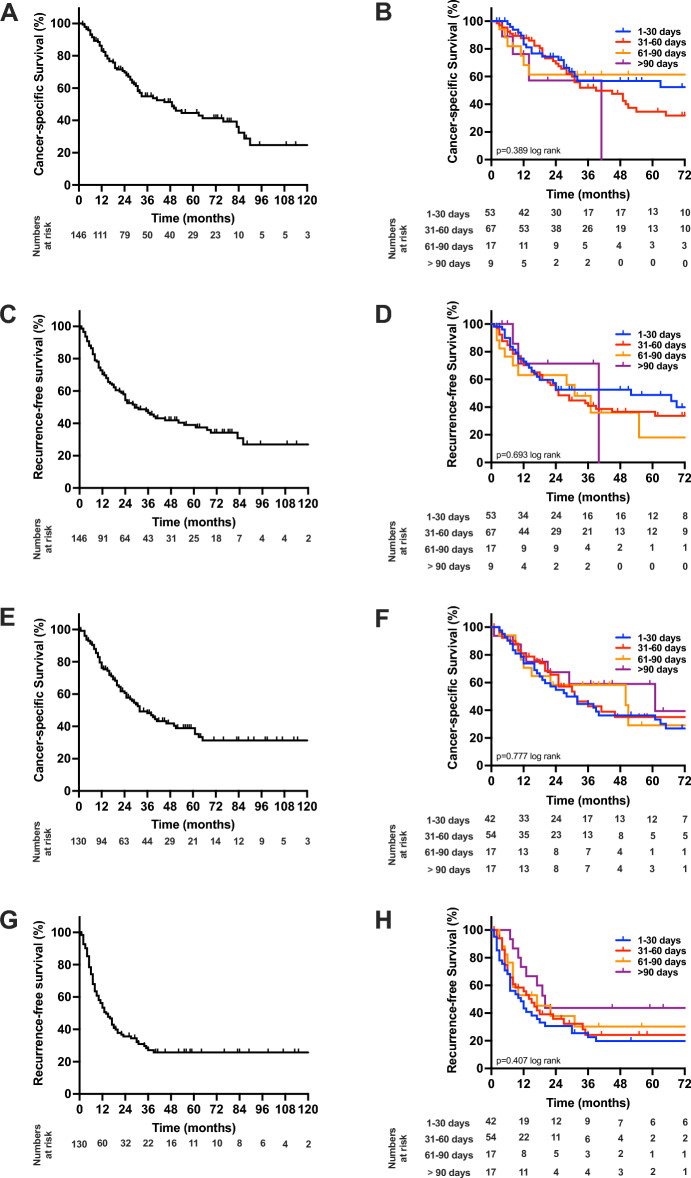
Oncological survival in cholangiocarcinoma stratified by time to surgery with respect to tumor type. (**A**) Cancer-specific in perihilar cholangiocarcinoma. The median CCS was 49 months. (**B**) Cancer-specific in perihilar cholangiocarcinoma stratified by time to surgery. The median CSS were 76 months (TTS 1–30 d), 39 months (TTS 31–60 d), 83 months (TTS 61–90 d) and 41 months (TTS > 90 d). (**C**) Recurrence-free survival in perihilar cholangiocarcinoma. The median RFS was 29 months. (**D**) Recurrence-free survival in perihilar cholangiocarcinoma stratified by time to surgery. The median RFS were 52 months (TTS 1–30 d), 25 months (TTS 31–60 d), 31 months (TTS 61–90 d) and 40 months TTS > 90 d). (**E**) Cancer-specific in intrahepatic cholangiocarcinoma. The median CCS was 32 months. (**F**) Cancer-specific in intrahepatic cholangiocarcinoma stratified by time to surgery. The median CSS were 28 months (TTS 1–30 d), 31 months (TTS 31–60 d), 50 months (TTS 61–90 d) and 61 months (TTS > 90 d). (**G**) Recurrence-free survival in intrahepatic cholangiocarcinoma. The median RFS was 13 months. (**H**) Recurrence-free survival in intrahepatic cholangiocarcinoma stratified by time to surgery. The median RFS were 11 months (TTS 1–30 d), 15 months (TTS 31–60 d), 17 months (TTS 61–90 d) and 19 months (TTS > 90 d). *CCS* cancer-specific survival, *RFS* recurrence-free survival.

We further conducted a survival analysis in different TTS groups for pCCA (Fig. 2A,C) and iCCA (Fig. 2E,G) separately to ensure the validity of our results in both tumor subtypes. Here, for pCCA, the median CSS were 76 months (TTS 1–30 d), 39 months (TTS 31–60 d), 83 months (TTS 61–90 d) and 41 months (TTS > 90 d; *p* = 0.389 log rank; Fig. 2B) and the median RFS were 52 months (TTS 1–30 d), 25 months (TTS 31–60 d), 31 months (TTS 61–90 d) and 40 months TTS > 90 d; *p* = 0.693 log rank; Fig. 2D). In iCCA, the median CSS were 28 months (TTS 1–30 d), 31 months (TTS 31–60 d), 50 months (TTS 61–90 d) and 61 months (TTS > 90 d; *p* = 0.777 log rank; Fig. 2F) and the median RFS were 11 months (TTS 1–30 d), 15 months (TTS 31–60 d), 17 months (TTS 61–90 d) and 19 months TTS > 90 d; *p* = 0.407 log rank; Fig. 2H).

### Univariate and multivariate cox regression analyses

Cox regression analyses were used to identify variables associated with CSS and RFS. For CSS, INR (*p* = 0.002), preoperative hemoglobin (*p* = 0.0002), CRP (*p* = 0.01), major hepatectomies (*p* = 0.049), PRBC (*p* = 0.001) as well as FFP (*p* = 0.001) transfusion, R1 status (*p* = 0.001), microvascular invasion (MVI, *p* = 0.001), lymphovascular invasion (LVI, *p* = 0.001), tumor grading (*p* = 0.001), pN category (*p* = 0.001), hospitalization (*p* = 0.011) and perioperative complications (*p* = 0.023) showed statistical significance in univariate analysis (Table 2). The above-mentioned variables were transferred into multivariable analysis, indicating in-hospital FFP transfusion (*p* = 0.03), LVI (*p* = 0.036), tumor grading (*p* = 0.012) and pN category (*p* = 0.006) to be independent prognostic factors for CSS (Table 3). TTS showed no statistical significance for this analysis in CCS. For RFS, a similar approach was conducted. In comparison to CCS, several parameters regarding tumor stage as well as a few preoperative blood parameters such as hemoglobin (*p* = 0.002), INR (*p* = 0.04), and R1 resection (*p* = 0.001) showed a statistical significance in univariate analysis. In addition, univariate cox regression analysis revealed that TTS has no quantitative impact on oncological outcome (*p* = 0.755). Multivariable cox regression analysis of the above-mentioned parameters revealed tumor entity (*p* = 0.001) and pN category (*p* = 0.001) being independent predictors for RFS (Table 3). In accordance with CCS, TTS showed no statistical significance in RFS.

**Table 3:** Multivariable analysis of cancer-specific and recurrence-free survival in cholangiocarcinoma.

	Recurrence-free survival (RFS)		Cancer-specific survival (CSS)	
	Relative risk (95% CI)	*P* value	Relative risk (95% CI)	*P* value
Tumor entity		**0.001**		
pCCA	1			
iCCA	2.46 (1.660–3.650)			
Platelets		0.094		
≤ 250				
> 250				
INR		0.079		0.073
≤ 1				
> 1				
Hemoglobin, g/dl		0.072		0.485
≤ 13				
> 13				
CRP, mg/l				0.390
≤ 10				
> 10				
Operative procedure		0.173		0.643
Monosegmentectomy + bisegmentectomy				
Hemihepatectomy				
Ext. hemihepetectomy + trisectionectomy				
others				
Monosegmentectomy + bisegmentectomy				
Hemihepatectomy				
In-hospital PRBC (qualitatively)		0.860		0.835
No				
Yes				
In-hospital FFP (qualitatively)		0.190		**0.030**
No			1	
Yes			1.77 (1.057–2.964)	
R1 resection		0.086		0.469
No				
Yes				
MVI		0.399		0.219
No				
Yes				
LVI		0.054		**0.036**
No			1	
Yes			1.70 (1.036–2.801)	
Tumor grading		0.143		**0.012**
G1/G2			1	
G3/G4			1.80 (1.141–2.840)	
pN category		**0.001**		**0.006**
N0	1		1	
N1	2.00 (1.342–2.993)		1.86 (1.195–2.878)	
Hospitalization, days		0.431		0.617
≤ 14				
> 14				
Perioperative complications				0.453
Clavien-Dindo I/II/IIIa				
Clavien-Dindo IIIb/IV				
Adjuvant therapy		0.928		
No				
Yes				

Relative risks are only provided for significant parameters.

*CSS* cancer-specific survival, *CI* confidence interval, *FFP* fresh frozen plasma, *pCCA* perihilar cholangiocarcinoma, *iCCA* intrahepatic cholangiocarcinoma, *LVI* lympho-vascular invasion, *MVI* microvascular invasion, *PRBC* packed red blood cells, *PVE* portal vein ligation, *RFS* recurrence-free survival, *UICC* Union for international cancer control.

Significant values are in bold.

Separate cox regression analysis for both tumor entities were also carried out. In multivariable analysis, hemoglobin (*p* = 0.013), in-hospital FFP (*p* = 0.001), MVI (*p* = 0.001), pN category (*p* = 0.005) and ICU time (*p* = 0.042) were independent prognostic factors for CCS in pCCA. For RFS, independent statistical differences were observed for hemoglobin (*p* = 0.003), in-hospital FFP (*p* = 0.018), LVI (*p* = 0.008) and pN category (*p* = 0.001) (supplementary table 3 and 5).

For iCCA, CRP (*p* = 0.009), R1 status (*p* = 0.058) and pN category (*p* = 0.001) were independent prognostic factors for CCS (supplementary table 4 and 6). For RFS, independent statistical differences were observed for operative procedure (*p* = 0.019), R1 status (*p* = 0.039) and pN category (*p* = 0.001) (Supplementary Table 4 and 6).

Both analyses showed no impact of TTS on oncological outcome in both pCCA (CSS, *p* = 0.389; RFS, *p* = 0.693) and iCCA (CSS, *p* = 0.777; RFS: *p* = 0.407).

## Discussion

Extended liver surgery remains the gold standard for patients with localized CCA being the only curative-intent treatment option for these patients. CCAs are highly aggressive and frequently diagnosed at advanced stages. Due to these factors and a difficult surgical treatment, CCAs are associated with poor oncological survival. As such, it is not only of utmost importance to accurately diagnose this type of cancer at early stages, but it also seems plausible to initiate therapy as soon as possible.

Therefore, we investigated the role TTS regarding oncological survival in both iCCA and pCCA patients undergoing curative-intent liver surgery. In a large European cohort, we demonstrated that TTS has no negative impact on RFS and CSS in univariate analysis. Remarkably, no major differences in perioperative characteristics were identified in individuals with different TTS intervals. Further, iCCA was significantly associated with reduced RFS in multivariable analyses. In addition, nodal status appears to be an independent risk factor for both RFS and CSS. In our analysis, in-hospital transfusion of FFPs, LVI and tumor grading were found to be independent prognostic factors for CSS.

Current available data investigating the role of TTS in surgically resected individuals with CCA is sparse, especially in the light of the COVID-19 pandemic. Ikemura et al. have investigated the impact of the COVID-19 pandemic on the frequency of endoscopic procedures in a Japanese center. Here, neither the number of ERCP cases was significantly reduced nor a significant reduction in the newly diagnosed pancreaticobiliary cancer was observed^14^. Interestingly, Ruys et al. conducted in 2014 a retrospective analysis comprising 209 patients with pCCA undergoing treatment and investigated whether therapeutic time frames impact clinical outcome^15^. Treatment time was defined as the time from the first clinical symptoms to final diagnosis of pCCA. This time was not associated with resectability, tumor stage or metastases. Only a small fraction of these patients (27% (56/209)) underwent resection, thus no meaningful analysis was possible regarding postoperative survival. Also, study was focussed on the time from symptoms to diagnosis in a referral center and explicitly not the time from diagnosis to surgery^15^. Hence, to the best of our knowledge our study is the first report in the literature to comprehensively analyze the role of the time frame from diagnosis to surgery in cholangiocarcinoma.

TTS has been investigated in other malignancies. Interestingly, a large systematic review demonstrated a worsened OS after each 4 weeks of delay to definitive surgery in bladder-, breast-, colon- and head/neck cancer^10^. Regarding other gastrointestinal carcinomas, a 2020 published study showed an improved OS in pancreatic adenocarcinoma if surgery was conducted within 6 weeks after time of diagnosis^16^. For gastric cancer on the other hand, a prolonged time to surgery seems to have no effect on OS according to a recent study^17^. In case of colorectal liver metastasis undergoing liver resection, a larger monocentric retrospective study displayed a worse OS for patients undergoing liver resection with a time to surgery longer than 6 months^18^. A part of this cohort underwent neoadjuvant chemotherapy, whereas in our study, patients with any preoperative treatment were excluded to reduce bias in the cohort.

Little literature-based guidance is available regarding the management of hepatobiliary cancers when surgery might not be possible due to a lag of resources. Bennett et al. recently reviewed different strategies compensating the delay in surgery due to the COVID-19 pandemic in patients with colorectal liver metastases, HCC, gallbladder cancer, iCCA and pCCA^19^. As surgery is usually the best treatment for iCCA and pCCA with respect to long-term survival, liver resection is highly recommended despite lagging resources in this guideline review. It is certainly interesting to speculate why our results do not indicate TTS to be a prognostic marker. Certainly, micro metastases or the onset of multifocal disease might be a problem especially in iCCA as these features are considered to be associated with a dismal prognosis^20^. In our department, patients with multifocal disease are still considered surgical candidates and notable proportion of patients with iCCA displayed more than one tumor nodule in our study cohort (40/130, 30.8%). Due to its anatomical location at the liver hilum, pCCA is known for the infiltration of major vessels which might progress during waiting time. Complex vascular reconstructions in pCCA are conducted in many large hepatobiliary centers, thus effect of local progression might also be detrimental with respect to TTS if aggressive vascular reconstruction is regularly carried out^21^. As vascular reconstructions are associated with an increase in postoperative morbidity and mortality, it is debatable whether prolonged TTS might result in a higher rate of postoperative complications. As patients displaying perioperative mortality were excluded from the analysis to ensure a focus on the pure oncological effect, our data does not allow to elaborate on this hypothesis but the rate of perioperative complications excluding mortality was not different among the TTS groups in our study. In an additional analysis, we also investigated the numbers of exploratory laparotomies without liver resection due to technical irresectablity or previously undetected distant metastases or peritoneal carcinomatosis. Of note, we were not able to observe a notable amount of more exploratory laparotomies in patients which were subject to longer TTS during the same study period which underlines our results.

Besides our primary observation of the impact of TTS on oncological outcome in individuals with CCA, we identified multiple prognostic factors which are in accordance with other available data. Nodal status is known risk factor in CCA with an even more pronounced effect in iCCA^22^. The same accounts for LVI^23,24^. Our group has recently reported on the prognostic value of FFP in surgically resected CCA while other transfusion characteristics e. g. PRBC were already extensively investigated^11,25,26^.

Perioperative complications were related to CCS in our univariate analysis and perioperative mortality is major factor in CCA as figures of up to 15% in selected subgroups have been reported^27,28^. Previous meta-analyses already demonstrated reduced hospitalization^29^ and complication rates^30^ in patients undergoing prehabilitation prior to major abdominal surgery. Especially in pCCA, preoperative drainage of the biliary system is often required to decompress cholestasis and treat preoperative infections and thus, delays surgery^31,32^. These studies indicate that detailed preoperative optimization is necessary to improve overall outcome in CCA and underline the need for randomized trials to investigate this problem. Our data suggest that a prolonged TTS (e.g. due to preoperative optimization in trial) does not necessarily impair long-term oncological outcome. Therefore, our results do support such studies from an oncological risk perspective for the individual participants.

To ensure validity of our results obtained for CCA in general, several subanalyses were conducted. Importantly, we assessed pCCA and iCCA separately regarding all analyses and found no impact of TTS on oncological outcome in either of the analyses. The same accounts for the inclusion of perioperative mortality as well as OS instead of CCS as primary outcome parameter of the study.

As with all retrospective analyses, our study has certainly limitations having to be considered when interpreting the results. Within the monocentric setting of our study, the data reflects the authors individual approach to CCA which might be different to clinical standards of other hepatobiliary centers. Our center regularly conducts arterial and portal venous resections in pCCA as well as complex surgical procedures in iCCA e.g. ALPPS (associating liver resection and portal vein ligation or for staged hepatectomy) resulting in a high surgical limit in terms of resectability which might influence our results. While the focus of our study was to investigate the influence of TTS in surgically treated patients, we are not able to report on patients dropping from surgical treatment plans due to progression during waiting time as only a small subset of patients was diagnosed in our hepatobiliary center and most of the TTS interval was based on the time from diagnosis to initially presentation to our hepatobiliary unit and not on the waiting time for surgery. Thus, patients becoming irresectable shortly after diagnosis or during diagnostic work-up were not included in this analysis. In addition, some of the liver volume modulation (in particular PVE) as well as biliary decompression strategies (especially in pCCA) might interfere with our data. However, to overcome this limitation to some extent we numerically analyzed the number of exploratory laparotomies during the study period and were not able to observe an effect of TTS.

Notwithstanding the mentioned limitations, we demonstrated that TTS does not influence CCS and RFS in patients with CCA who underwent liver resection in curative-intent. Further, our results suggest prehabilitation as important measure to improve short- and long-term outcomes in surgical candidates with CCA.

### Supplementary Information

Supplementary Tables.

## Data Availability

All relevant data were reported within the manuscript. Further supporting data will be provided upon written request addressed to the corresponding author.

## Abbreviations

ALPPS: Associating liver partition and portal vein ligation for staged hepatectomy
ALT: Alanine aminotransferase
ASA: American society of anesthesiologists
AST: Aspartate aminotransferase
BMI: Body mass index
CCA: Cholangiocellular carcinoma
CI: Confidence interval
CRP: C reactive protein
CSS: Cancer-specific survival
CT: Computed tomography
COVID-19: Coronavirus disease 2019
dCCA: Distal cholangiocellular carcinoma
FFP: Fresh frozen plasma
FLR: Future liver remnant
GGT: Gamma glutamyltransferase
HCC: Hepatocellular carcinoma
iCCA: Intrahepatic cholangiocarcinoma
INR: International normalized ratio
LVI: Lympho-vascular invasion
MVI: Microvascular invasion
OS: Overall survival
pCCA: Perihilar cholangiocarcinoma
PRBC: Packed red blood cells
RWTH: Rheinisch-Westfälische Technische Hochschule
TTS: Time to surgery
UICC: Union for international cancer control
