# Supplementary material for: Time to surgery is not an oncological risk factor in patients with cholangiocarcinoma undergoing curative-intent liver surgery
Source: Sci Rep. 2024 Jan 18;14:1644. doi: 10.1038/s41598-023-50842-6 (PMC10796920; doi:10.1038/s41598-023-50842-6)
Supplement: Supplementary file 1 — Supplementary Tables. [file 41598_2023_50842_MOESM1_ESM.docx]

**SUPPLEMENTARY DATA**

**Supplementary table 1: Patients’ characteristics (perihilar cholangiocarcinoma)**

| **Demographics** | **pCCA (n=146)** | **Time to surgery subanalysis** | | | | **p value** |
| --- | --- | --- | --- | --- | --- | --- |
|  |  | **1 – 30 days**  **(n=53)** | **31 – 60 days**  **(n=67)** | **61 – 90 days**  **(n=17)** | **>90 days**  **(n=9)** |  |
| Gender, m/f (%) | 95 (65.1) / 51 (34.9) | 31 (58.5) / 22 (41.5) | 46 (68.7) / 21 (31.3) | 13 (76.5) / 4 (23.5) | 5 (55.6) / 4 (44.4) | .437 |
| Age (years) | 68 ± 10 | 68 ± 11 | 69 ± 10 | 65 _­_± 8 | 69 ± 8 | .433 |
| BMI (kg/m^2^) | 25 ± 5 | 25 ± 5 | 25 ± 4 | 25 ± 4 | 25 ± 7 | .735 |
| Portal vein embolization, n (%) | 53 (36.3) | 8 (15.1) | 27 (40.3) | 11 (64.7) | 7 (77.8) | **.001** |
| ASA, n (%) |  |  |  |  |  | .656 |
| I | 5 (3.4) | 1 (1.9) | 4 (6.0) | 0 | 0 |  |
| II | 52 (35.6) | 22 (41.5) | 20 (29.9) | 8 (47.1) | 2 (22.2) |  |
| III | 79 (54.1) | 26 (49.1) | 38 (56.7) | 9 (52.9) | 6 (66.7) |  |
| IV | 10 (6.8) | 4 (7.5) | 5 (7.5) | 0 | 1 (11.1) |  |
| V | 0 | 0 | 0 | 0 | 0 |  |
| Preoperative Chemotherapy | 0 | 0 | 0 | 0 | 0 | n.a. |
| Future liver remnant modulation |  |  |  |  |  |  |
| endoscopic stenting | 104 (71.2) | 35 (66) | 48 (71.6) | 12 (70.6) | 9 (100) |  |
| Percutaneous transhepatic biliary drainage | 35 (24) | 10 (18.5) | 19 (28.4) | 6 (35.3) | 0 |  |
| Exploratory laparotomies during the same study period, n (%)** | n.a. | n.a. | n.a. | n.a. | n.a. | n.a. |
| **Clinical chemistry (preoperative)** |  |  |  |  |  |  |
| AST (U/l) | 47 ± 254 | 51 ± 389 | 48 ± 139 | 45 ± 37 | 40 ± 21 | .617 |
| GGT (U/l) | 461 ± 563 | 396 ± 659 | 525 ± 507 | 441 ± 595 | 517 ± 321 | .858 |
| Total bilirubin (mg/dl) | 1.1 ± 3.1 | 1.7 ± 4.1 | 1.2 ± 2.4 | 0.8 ± 0.9 | 0.6 ± 0.7 | **.026** |
| Hemoglobin (g/dl) | 12.2 ± 1.7 | 12.1 ± 1.6 | 12.2 ± 1.8 | 12.5 ± 1.5 | 13.5 ± 1.6 | .681 |
| Platelet count (/nl) | 294 ± 136 | 339 ± 132 | 276 ± 145 | 296 ± 96 | 247 ± 150 | **.044** |
| INR | 1 ± 2.23 | 1.03 ± 0.14 | 1.02 ± 0.11 | 1.05 ± 6.68 | 1.02 ± 0.48 | .751 |
| Prothrombin time (%) | 96 ± 17 | 95 ± 18 | 97 ± 15 | 95 ± 15 | 97 ± 27 | .917 |
| CRP (mg/l) | 12 ± 41 | 14 ± 41 | 12 ± 35 | 15 ± 64 | 16 ± 15 | .530 |
| **Operative Data** |  |  |  |  |  |  |
| Operative time (minutes) | 425 ± 98 | 420 ± 100 | 423 ± 101 | 454 ± 86 | 480 ± 64 | .135 |
| Operative procedure, n (%) |  |  |  |  |  | .440 |
| Atypical | 0 | 0 | 0 | 0 | 0 |  |
| Monosegmentectomy | 0 | 0 | 0 | 0 | 0 |  |
| Bisegmentectomy | 0 | 0 | 0 | 0 | 0 |  |
| Hemihepatectomy | 39 (26.7) | 18 (33.9) | 14 (20.9) | 4 (23.6) | 3 (33.3) |  |
| Extended hemihepatectomy^#^ | 63 (43.1) | 25 (47.2) | 29 (43.3) | 5 (35.3) | 4 (44.4) |  |
| Trisectionectomy | 29 (19.9) | 5 (9.5) | 17 (25.4) | 6 (35.3) | 1 (11.1) |  |
| Hepatoduodenoectomy^†^ | 8 (5.5) | 1 (1.9) | 5 (7.5) | 1 (5.9) | 1 (11.1) |  |
| ALPPS | 1 (0.7) | 1 (1.9) | 0 | 0 | 0 |  |
| other | 6 (4.1) | 3 (5.7) | 2 (3) | 1 (5.9) | 0 |  |
| Laparoscopic resection, n (%) | 0 | 0 | 0 | 0 | 0 | n.a. |
| Intraoperative PRBC, n (%) | 71 (48.6) | 27 (50.9) | 30 (44.8) | 9 (52.9) | 5 (55.6) | .645 |
| Intraoperative FFP, n (%) | 80 (54.8) | 28 (52.8) | 36 (53.7) | 12 (70.6) | 4 (44.4) | .826 |
| Intraoperative platelets, n (%) | 1 (0.7) | 0 | 0 | 0 | 1 (11.1) | **.002** |
| **Pathological examination** |  |  |  |  |  |  |
| R1 resection, n (%) | 27 (18.5) | 7 (13.2) | 12 (17.9) | 7 (41.2) | 1 (11.1) | .116 |
| pN category, n (%) |  |  |  |  |  | .244 |
| N0 | 83 (56.8) | 35 (66) | 37 (55.2) | 7 (41.2) | 4 (44.4) |  |
| N1 | 63 (43.2) | 18 (34) | 30 (44.8) | 10 (58.8) | 5 (55.6) |  |
| Tumor grading, n (%) |  |  |  |  |  | .851 |
| G1 | 6 (4.1) | 2 (3.8) | 3 (4.5) | 1 (6.3) | 0 |  |
| G2 | 100 (68.5) | 36 (67.9) | 43 (65.2) | 13 (81.3) | 8 (88.9) |  |
| G3 | 36 (24.7) | 15 (28.3) | 18 (27.2) | 2 (12.5) | 1 (11.1) |  |
| G4 | 1 (0.7) | 0 | 1 (1.5) | 0 | 0 |  |
| MVI, n (%) | 44 (30.6) | 18 (34) | 19 (29.2) | 6 (35.3) | 1 (11.1) | .441 |
| LVI, n (%) | 33 (22.6) | 15 (28.3) | 12 (18.8) | 6 (35.3) | 0 | .109 |
| pT category n (%) |  |  |  |  |  | .064 |
| 1 | 13 (8.9) | 5 (9.4) | 3 (4.5) | 3 (17.6) | 2 (22.2) |  |
| 2 | 85 (58.3) | 26 (49.1) | 44 (65.7) | 10 (58.8) | 5 (55.5) |  |
| 3 | 36 (24.7) | 21 (39.6) | 13 (19.4) | 1 (5.9) | 1 (11.1) |  |
| 4 | 12 (8.2) | 1 (1.9) | 7 (10.4) | 3 (17.6) | 1 (11.1) |  |
| **Postoperative Data** |  |  |  |  |  |  |
| Intensive care, days | 1 ± 9 | 1 ± 2 | 1 ± 13 | 1 ± 9 | 3 ± 2 | .242 |
| Hospitalization, days | 19 ± 29 | 16 ± 14 | 21 ± 39 | 22 ± 22 | 31 ± 20 | .345 |
| Postoperative complications, n (%) |  |  |  |  |  | .262 |
| No complications | 27 (18.5) | 14 (26.4) | 11 (16.4) | 0 | 2 (22.2) |  |
| Clavien-Dindo I | 9 (6.2) | 2 (3.8) | 4 (6) | 2 (11.8) | 1 (11.1) |  |
| Clavien-Dindo II | 36 (24.7) | 14 (26.4) | 19 (28.4) | 3 (17.6) | 0 |  |
| Clavien-Dindo IIIa | 31 (21.2) | 9 (17) | 12 (17.9) | 7 (41.2) | 3 (33.3) |  |
| Clavien-Dindo IIIb | 30 (20.5) | 12 (22.6) | 13 (19.4) | 2 (11.8) | 3 (33.3) |  |
| Clavien-Dindo IVa | 8 (5.5) | 2 (3.8) | 5 (7.5) | 1 (5.9) | 0 |  |
| Clavien-Dindo IVb | 5 (3.4) | 0 | 3 (4.5) | 2 (11.8) | 0 |  |
| Clavien-Dindo V | 0 | 0 | 0 | 0 | 0 |  |
| **Oncologic Data*** |  |  |  |  |  |  |
| Adjuvant chemotherapy, n (%) | 47 (32.4) | 20 (37.7) | 22 (32.8) | 5 (31.3) | 0 | .171 |
| Recurrence, n (%) | 78 (53.4) | 25 (48.1) | 40 (60.6) | 10 (58.8) | 3 (33.3) | .308 |
| Median RFS, months (95% CI) | 29 (19-39) | 52 (0.3-104) | 25 (17-33) | 31 (20-43) | 31 (18-44)*** | .693 |
| Median CSS, months (95% CI) | 49 (32-66) | 74 (57-90)*** | 39 (21-57) | 54 (35-74)*** | 28 (14-41)*** | .389 |
| Median OS, months (95% CI) | 33 (23-43) | 51 (25-77) | 33 (23-43) | 43 (10-25)*** | 22 (10-33)*** | .227 |

*Data presented as mean and standard deviation if not noted otherwise. *Data presented as median and interquartile range. #Right or left hepatectomy were considered to be extended hepatectomies if the middle hepatic vein was removed and the resection was extended into the segments IV or V/VIII, respectively. Procedures were defined as hepatoduodenoectomy if a major liver resections was combined with the concomitant resection of the pancreatic head. ALPPS, Associating liver partition and portal vein ligation for staged hepatectomy; ASA, American society of anesthesiologists classification; AST, aspartate aminotransferase; BMI, body mass index; CSS, cancer-specific survival; EBD, endoscopic biliary drainage; FFP, fresh frozen plasma; pCCA, perihilar cholangiocarcinoma; GGT, gamma glutamyltransferase; iCCA; intrahepatic cholangiocarcinoma; INR, international normalized ratio; LVI, lympho-vascular invasion; MVI, microvascular invasion;* *n./a., not applicable; OS, overall survival. PRBC, packed red blood cells; RFS, disease free survival; UICC, Union for international cancer control.* **This data was not included in statistical analysis. ***Data presented as mean.

**Supplementary table 2: Patients’ characteristics (intrahepatic cholangiocarcinoma)**

| **Demographics** | **iCCA (n=130)** | **Time to surgery subanalysis** | | | | **p value** |
| --- | --- | --- | --- | --- | --- | --- |
|  |  | **1 – 30 days**  **(n=42)** | **31 – 60 days**  **(n=54)** | **61 – 90 days**  **(n=17)** | **>90 days**  **(n=17)** |  |
| Gender, m/f (%) | 58 (44.6) / 72 (55.4) | 15 (35.7) / 27 (64.3) | 28 (51.9) / 26 (48.1) | 8 (47.1) / 9 (52.9) | 7 (41.2) / 10 (58.8) | .455 |
| Age (years) | 66 ± 11 | 62 ± 11 | 71 ± 11 | 70 _­_± 10 | 65 ± 10 | **.031** |
| BMI (kg/m^2^) | 26 ± 5 | 26 ± 4 | 26 ± 5 | 27 ± 6 | 27 ± 5 | .576 |
| Portal vein embolization, n (%) | 8 (6.2) | 1 (2.4) | 2 (3.7) | 2 (11.8) | 3 (17.6) | .093 |
| ASA, n (%) |  |  |  |  |  | .389 |
| I | 4 (3.1) | 2 (4.9) | 1 (1.9) | 1 (5.9) | 0 |  |
| II | 51 (39.5) | 21 (51.2) | 17 (31.5) | 6 (35.3) | 7 (41.2) |  |
| III | 68 (52.7) | 16 (39) | 34 (63) | 10 (58.8) | 8 (47.1) |  |
| IV | 6 (4.7) | 2 (4.9) | 2 (3.7) | 0 | 2 (11.8) |  |
| V | 0 | 0 | 0 | 0 | 0 |  |
| Preoperative Chemotherapy | 0 | 0 | 0 | 0 | 0 | n.a. |
| Future liver remnant modulation |  |  |  |  |  |  |
| endoscopic stenting | 13 (10) | 5 (11.9) | 6 (11.1) | 0 | 2 (11.8) |  |
| Percutaneous transhepatic biliary drainage | 0 | 0 | 0 | 0 | 0 |  |
| ALPPS (step one) | 11 (8.5) | 3 (7.1) | 4 (7.4) | 3 (17.6) | 1 (5.9) |  |
| Portal vein embolization, n (%) | See above | See above | See above | See above | See above | See above |
| Exploratory laparotomies during the same study period, n (%)** | n.a. | n.a. | n.a. | n.a. | n.a. | n.a. |
| **Clinical chemistry (preoperative)** |  |  |  |  |  |  |
| AST (U/l) | 35 ± 60 | 37 ± 52 | 33 ± 77 | 38 ± 16 | 27 ± 47 | .190 |
| GGT (U/l) | 100 ± 348 | 220 ± 368 | 82 ± 389 | 162 ± 141 | 63 ± 282 | .103 |
| Total bilirubin (mg/dl) | 0.5 ± 3.4 | 0.5 ± 5.8 | 0.5 ± 1 | 0.5 ± 0.3 | 0.5 ± 0.4 | .961 |
| Hemoglobin (g/dl) | 13.3 ± 1.5 | 13.2 ± 1.5 | 13.3 ± 1.5 | 13.7 ± 1.4 | 12.6 ± 1.8 | .651 |
| Platelet count (/nl) | 254 ± 88 | 285 ± 99 | 230 ± 89 | 277 ± 59 | 256 ± 52 | .111 |
| INR | 1 ± 0.1 | 1 ± 0.1 | 1 ± 0.11 | 1 ± 0.1 | 1 ± 0.1 | .614 |
| Prothrombin time (%) | 100 ± 14 | 100 ± 12 | 100 ± 16 | 104 ± 10 | 100 ± 13 | .585 |
| CRP (mg/l) | 8 ± 35 | 10 ± 27 | 5 ± 47 | 7 ± 16 | 8 ± 13 | .371 |
| **Operative Data** |  |  |  |  |  |  |
| Operative time (minutes) | 295 ± 109 | 289 ± 109 | 295 ± 107 | 289 ± 117 | 333 ± 120 | .941 |
| Operative procedure, n (%) |  |  |  |  |  | .733 |
| Atypical | 14 (10.8) | 3 (7.1) | 7 (13) | 2 (11.8) | 2 (11.8) |  |
| Monosegmentectomy | 1 (0.8) | 0 | 0 | 1 (5.9) | 0 |  |
| Bisegmentectomy | 12 (9.3) | 4 (9.5) | 6 (11.2) | 0 | 2 (11.8) |  |
| Hemihepatectomy | 48 (36.9) | 20 (47.6) | 18 (33.4) | 6 (35.4) | 4 (23.5) |  |
| Extended hemihepatectomy^#^ | 24 (18.4) | 6 (14.3) | 9 (16.8) | 4 (23.5) | 5 (29.4) |  |
| Trisectionectomy | 14 (10.8) | 4 (9.6) | 7 (13.1) | 1 (5.9) | 2 (11.8) |  |
| Hepatoduodenoectomy^†^ | 0 | 0 | 0 | 0 | 0 |  |
| ALPPS | 11 (8.5) | 3 (7.1) | 4 (7.4) | 3 (17.3) | 1 (5.9) |  |
| other | 6 (4.6) | 2 (4.8) | 3 (5.6) | 0 | 1 (5.9) |  |
| Laparoscopic resection, n (%) | 15 (19.7) | 3 (16.7) | 8 (24.2) | 1 (7.1) | 3 (27.3) | .508 |
| Intraoperative PRBC, n (%) | 30 (23.1) | 13 (31) | 12 (22.2) | 3 (17.6) | 2 (11.8) | .420 |
| Intraoperative FFP, n (%) | 40 (30.8) | 15 (35.7) | 17 (31.5) | 4 (23.5) | 4 (23.5) | .588 |
| Intraoperative platelets, n (%) | 1 (0.8) | 1 (2.4) | 0 | 0 | 0 | .553 |
| **Pathological examination** |  |  |  |  |  |  |
| R1 resection, n (%) | 10 (7.8) | 5 (12.2) | 2 (3.7) | 2 (11.8) | 1 (5.9) | .357 |
| pN category, n (%) |  |  |  |  |  | .836 |
| N0 | 78 (65.5) | 27 (65.9) | 32 (66.7) | 9 (56.3) | 10 (71.4) |  |
| N1 | 41 (34.5) | 14 (34.1) | 16 (33.3) | 7 (43.8) | 4 (28.6) |  |
| Tumor grading, n (%) |  |  |  |  |  | .**041** |
| G1 | 1 (0.8) | 0 | 0 | 0 | 1 (6.7) |  |
| G2 | 83 (68) | 34 (85) | 32 (64) | 9 (52.9) | 8 (53.3) |  |
| G3 | 34 (27.8) | 6 (15) | 16 (32) | 7 (41.2) | 5 (33.3) |  |
| G4 | 4 (3.3) | 0 | 2 (4) | 1 (5.9) | 1 (6.7) |  |
| MVI, n (%) | 47 (37.6) | 16 (40) | 21 (40.4) | 5 (29.4) | 5 (31.3) | .794 |
| LVI, n (%) | 22 (18.2) | 7 (17.9) | 10 (20.4) | 3 (17.6) | 2 (12.5) | .916 |
| pT category n (%) |  |  |  |  |  | .940 |
| 1 | 51 (39.2) | 16 (38.1) | 18 (33.3) | 7 (41.2) | 10 (58.8) |  |
| 2 | 51 (39.4) | 18 (42.8) | 23 (42.6) | 7 (41.2) | 3 (17.7) |  |
| 3 | 18 (13.8) | 6 (14.3) | 8 (14.8) | 1 (5.9) | 3 (17.6) |  |
| 4 | 10 (7.7) | 2 (4.8) | 5 (9.3) | 2 (23.5) | 1 (5.9) |  |
| **Postoperative Data** |  |  |  |  |  |  |
| Intensive care, days | 1 ± 2 | 1 ± 1 | 1 ± 2 | 1 ± 2 | 1 ± 1 | .563 |
| Hospitalization, days | 12 ± 18 | 12 ± 15 | 13 ± 17 | 16 ± 28 | 12 ± 11 | .808 |
| Postoperative complications, n (%) |  |  |  |  |  | .513 |
| No complications | 52 (40) | 16 (38.1) | 21 (38.9) | 6 (35.3) | 9 (52.9) |  |
| Clavien-Dindo I | 6 (4.6) | 3 (7.1) | 2 (3.7) | 0 | 1 (5.9) |  |
| Clavien-Dindo II | 29 (22.3) | 9 (21.4) | 14 (25.9) | 3 (17.6) | 3 (17.6) |  |
| Clavien-Dindo IIIa | 29 (22.3) | 8 (19) | 13 (24.1) | 5 (29.4) | 3 (17.6) |  |
| Clavien-Dindo IIIb | 8 (6.2) | 2 (4.8) | 2 (3.7) | 3 (17.6) | 1 (5.9) |  |
| Clavien-Dindo IVa | 6 (4.6) | 4 (9.5) | 2 (3.7) | 0 | 0 |  |
| Clavien-Dindo IVb | 0 | 0 | 0 | 0 | 0 |  |
| Clavien-Dindo V | 0 | 0 | 0 | 0 | 0 |  |
| **Oncologic Data*** |  |  |  |  |  |  |
| Adjuvant chemotherapy, n (%) | 46 (35.4) | 19 (45.2) | 12 (22.2) | 8 (47.1) | 7 (41.2) | **.068** |
| Recurrence, n (%) | 82 (64.6) | 32 (78) | 30 (57.7) | 11 (64.7) | 9 (52.9) | .149 |
| Median RFS, months (95% CI) | 13 (9-17) | 11 (5-17) | 15 (10-21) | 17 (2-32) | 19 (14-24) | .407 |
| Median CSS, months (95% CI) | 32 (23-41) | 28 (16-40) | 31 (18-44) | 51 (28-74)*** | 61 (3-119) | .777 |
| Median OS, months (95% CI) | 30 (22-39) | 28 (16-40) | 25 (15-35) | 51 (28-74)*** | 59 (6-112) | .613 |

*Data presented as mean and standard deviation if not noted otherwise. *Data presented as median and interquartile range. #Right or left hepatectomy were considered to be extended hepatectomies if the middle hepatic vein was removed and the resection was extended into the segments IV or V/VIII, respectively. Procedures were defined as hepatoduodenoectomy if a major liver resections was combined with the concomitant resection of the pancreatic head. ALPPS, Associating liver partition and portal vein ligation for staged hepatectomy; ASA, American society of anesthesiologists classification; AST, aspartate aminotransferase; BMI, body mass index; CSS, cancer-specific survival; EBD, endoscopic biliary drainage; FFP, fresh frozen plasma; pCCA, perihilar cholangiocarcinoma; GGT, gamma glutamyltransferase; iCCA; intrahepatic cholangiocarcinoma; INR, international normalized ratio; LVI, lympho-vascular invasion; MVI, microvascular invasion;* *n./a., not applicable; OS, overall survival; PRBC, packed red blood cells; RFS, disease free survival; UICC, Union for international cancer control.* **This data was not included in statistical analysis. ***Data presented as mean.

**Supplementary table 3:** Univariable analysis of recurrence-free and cancer-specific survival in perihilar cholangiocarcinoma

|  |  | ***Recurrence-free survival (RFS)*** | | |  | ***Cancer-specific survival (CSS)*** | | |
| --- | --- | --- | --- | --- | --- | --- | --- | --- |
|  | **n** | **Median RFS, m (95% CI)** | **Relative risk (95% CI)/HR** | ***P* value** |  | **Median CSS, m (95% CI)** | **Relative risk (95% CI)/HR** | ***P* value** |
| **Sex** |  |  |  | .731 |  |  |  | .544 |
| Male | 95 | 31 (17-45) |  |  |  | 49 (10-88) |  |  |
| Female | 51 | 29 (12-46) |  |  |  | 45 (18-73) |  |  |
| **Age, years** |  |  |  | .439 |  |  |  | .580 |
| ≤ 65 | 61 | 25 (5-45) |  |  |  | 51 (22-81) |  |  |
| > 65 | 83 | 29 (10-48) |  |  |  | 45 (23-67) |  |  |
| **BMI, kg/m^2^** |  |  |  | .193 |  |  |  | .684 |
| ≤ 25 | 70 | 40 (11-69) |  |  |  | 49 (25-73) |  |  |
| > 25 | 74 | 24 (12-36) |  |  |  | 39 (17-62) |  |  |
| **PVE** |  |  |  | .998 |  |  |  | .723 |
| No | 91 | 25 (13-37) |  |  |  | 54 (4-104) |  |  |
| Yes | 53 | 37 (20-54) |  |  |  | 45 (27-63) |  |  |
| **ASA** |  |  |  | .467 |  |  |  | .232 |
| I / II | 56 | 36 (1-71) |  |  |  | 54 (36-72) |  |  |
| III / IV | 88 | 28 (21-35) |  |  |  | 33 (18-48) |  |  |
| **Time to surgery**  <30  30-60  60-90  >90  **AST, U/l**  ≤ 40  > 40 | 53  67  17  9  54  89 | 64 (47-80)***  25 (17-33)  31 (19-43)  31 (18-44)***  40 (14-66)  25 (11-39) |  | .693  .138 |  | 74 (57-90)***  39 (21-57)  54 (35-74)***  28 (14-41)***  49 (20-78)  49 (26-72) |  | .389  .635 |
| **GGT, U/l**  ≤ 100  > 100  **Bilirubin, mg/dl**  ≤ 1  > 1 | 32  106  67  77 | 45 (4-86)  25 (15-35)  37 (20-54)  24 (10-38) |  | .852  .126 |  | 65 (20-110)  45 (27-63)  54 (6-102)  45 (26-64) |  | .413  .294 |
| **Platelet count, 1/nl**  ≤ 250  > 250 | 48  96 | 29 (11-47)  35 (19-51) |  | .632 |  | 45 (27-63)  51 (18-84) |  | .781 |
| **INR**  ≤ 1  > 1 | 54  89 | 61 (16-106)  24 (13-36) | 1  1.607 (0.998-2.588) | .**047** |  | 76 (40-112)  31 (21-42) | 1  1.837 (1.090-3.097) | .**02** |
| **Hemoglobin, g/dl** |  |  |  | **.001** |  |  |  | **.001** |
| ≤ 12 | 66 | 15 (8-23) | 1 |  |  | 25 (16-35) | 1 |  |
| > 12 | 78 | 69 (30-108) | 0.602 (0.244-0.608) |  |  | 83 (51-116) | 0.435 (0.268-0.706) |  |
| **CRP, mg/l** |  |  |  | .159 |  |  |  | .804 |
| ≤ 10 | 58 | 24 (15-33) |  |  |  | 41 (7-75) |  |  |
| > 10 | 82 | 18 (11-26) |  |  |  | 50 (35-65) |  |  |
| **Operative time, min** |  |  |  | **.008** |  |  |  | **.033** |
| ≤ 360 | 37 | 86 (24-148) | 1 |  |  | 87 (79-95) | 1 |  |
| > 360 | 107 | 25 (18-32) | 2.146 (1.196-3.849) |  |  | 39 (24-54) | 1.887 (1.039-3.425) |  |
| **Operative procedure** |  |  |  | .914 |  |  |  | .635 |
| Monosegmentectomy + bisegmentectomy  Hemihepatectomy | 0  39 | 24 (14-34) |  |  |  | 39 (12-66) |  |  |
| Ext. Hemihepetectomy + Trisectionectomy | 101 | 37 (17-57) |  |  |  | 50 (22-79) |  |  |
| others | 4 | 25 (22-28) |  |  |  | 27 (17-37) |  |  |
| **In-hospital PRBC (qualitatively)** |  |  |  | .**001** |  |  |  | .**001** |
| No | 74 | 67 (33-101) | 1 |  |  | 84 (72-96) | 1 |  |
| Yes | 70 | 17 (9-25) | 2.146 (1.364-3.375) |  |  | 28 (18-38) | 2.641 (1.620-4.306) |  |
| **In-hospital FFP (qualitatively)** |  |  |  | **.001** |  |  |  | .**001** |
| No | 65 | 67 (18-116) | 1 |  |  | 90 (76-104) | 1 |  |
| Yes | 79 | 17 (3-31) | 2.168 (1.357-3.465) |  |  | 30 (22-38) | 3.084 (1.8-5.287) |  |
| **R1 resection** |  |  |  | **.001** |  |  |  | .**001** |
| No | 117 | 40 (16-65) | 1 |  |  | 63 (31-95) | 1 |  |
| Yes | 27 | 12 (5-19) | 2.485 (1.480-4.175) |  |  | 23 (5-41) | 2.655 (1.529-4.609) |  |
| **MVI** |  |  |  | **.001** |  |  |  | .**001** |
| No | 96 | 55 (24-87) | 1 |  |  | 76 (40-112) | 1 |  |
| Yes | 44 | 15 (5-25) | 2.542 (1.6-4.041) |  |  | 23 (13-33) | 2.846 (1.748-4.633) |  |
| **LVI** |  |  |  | .**001** |  |  |  | .**001** |
| No | 105 | 52 (26-78) | 1 |  |  | 65 (37-93) | 1 |  |
| Yes | 33 | 15 (8-23) | 2.797 (1.693-4.621) |  |  | 19 (13-25) | 2.688 (1.560-4.630) |  |
| **Tumor grading** |  |  |  | **.001** |  |  |  | .**001** |
| G1 / G2 | 105 | 52 (23-81) | 1 |  |  | 63 (32-94) | 1 |  |
| G3 / G4 | 34 | 16 (6-26) | 2.6 (1.575-4.29) |  |  | 26 (13-39) | 2.575 (1.497-4.429) |  |
| **pN category** |  |  |  | .**001** |  |  |  | .**001** |
| N0 | 82 | 67 (36-98) | 1 |  |  | 84 (43-125) | 1 |  |
| N1 | 62 | 15 (9-22) | 2.579 (1.64-4.054) |  |  | 25 (17-33) | 2.605 (1.612-4.210) |  |
| **ICU time, days** |  |  |  | **.030** |  |  |  | **.011** |
| ≤ 1 | 91 | 45 (22-68) | 1 |  |  | 63 (34-93) | 1 |  |
| > 1 | 53 | 24 (14-34) | 1.64 (1.04-2.588) |  |  | 26 (17-35) | 1.843 (1.136-2.991) |  |
| **Hospitalization, days** |  |  |  | **.037** |  |  |  | **.016** |
| ≤ 14 | 57 | 52 (19-85) | 1 |  |  | 76 (48-104) | 1 |  |
| > 14 | 83 | 24 (15-33) | 1.641 (1.022-2.634) |  |  | 31 (26-36) | 1.857 (1.108-3.112) |  |
| **Perioperative complications** |  |  |  | .402 |  |  |  | .138 |
| Clavien-Dindo I/II/IIIa | 102 | 31 (12-50) |  |  |  | 51 (17-85) |  |  |
| Clavien-Dindo IIIb/IV | 42 | 24 (6-42) |  |  |  | 32 (22-42) |  |  |
| **Adjuvant therapy** |  |  |  | **.037** |  |  |  | .805 |
| No | 98 | 45 (13-77) | 1 |  |  | 50 (21-79) |  |  |
| Yes | 45 | 19 (9-29) | 0.605 (0.374-0.980) |  |  | 39 (22-56) |  |  |

*Various parameters are associated with cancer-specific or recurrence -free survival. ASA, American society of anesthesiologists classification; AST, aspartate aminotransferase; BMI, body mass index; CSS, cancer-specific survival; FFP, fresh frozen plasma; pCCA, perihilar cholangiocarcinoma; GGT, gamma glutamyltransferase; iCCA; intrahepatic cholangiocarcinoma; INR, international normalized ratio; LVI, lympho-vascular invasion; MVI, microvascular invasion; PRBC, packed red blood cells; PVE, portal vein ligation. RFS, recurrence-free survival; UICC, Union for international cancer control.*

******Data presented as mean.

**Supplementary table 4:** Univariable analysis of recurrence-free and cancer-specific survival in intrahepatic cholangiocarcinoma

|  |  | ***Recurrence-free survival (RFS)*** | | |  | ***Cancer-specific survival (CSS)*** | | |
| --- | --- | --- | --- | --- | --- | --- | --- | --- |
|  | **n** | **Median RFS, m (95% CI)** | **Relative risk (95% CI)/HR** | ***P* value** |  | **Median CSS, m (95% CI)** | **Relative risk (95% CI)/HR** | ***P* value** |
| **Sex** |  |  |  | .725 |  |  |  | .149 |
| Male | 57 | 12 (8-16) |  |  |  | 24 (12-36) |  |  |
| Female | 70 | 17 (12-22) |  |  |  | 41 (19-63) |  |  |
| **Age, years** |  |  |  | .427 |  |  |  | .872 |
| ≤ 65 | 63 | 13 (8-19) |  |  |  | 32 (20-44) |  |  |
| > 65 | 67 | 17 (8-26) |  |  |  | 32 (15-48) |  |  |
| **BMI, kg/m^2^** |  |  |  | .707 |  |  |  | .731 |
| ≤ 25 | 57 | 12 (6-18) |  |  |  | 32 (20-44) |  |  |
| > 25 | 72 | 15 (10-20) |  |  |  | 30 (16-44) |  |  |
| **PVE** |  |  |  | .309 |  |  |  | .603 |
| No | 119 | 15 (10-20) |  |  |  | 32 (23-41) |  |  |
| Yes | 8 | 12 (7-17) |  |  |  | 22 (7-37) |  |  |
| **ASA** |  |  |  | .424 |  |  |  | .325 |
| I / II | 55 | 17 (12-22) |  |  |  | 38 (18-58) |  |  |
| III / IV | 74 | 11 (6-16) |  |  |  | 30 (18-42) |  |  |
| **Time to surgery**  <30  30-60  60-90  >90  **AST, U/l**  ≤ 40  > 40 | 42  54  17  17  82  47 | 11 (5-17)  15 (10-21)  17 (2-32)  19 (14-24)  17 (13-21)  10 (6-14) |  | .407  .190 |  | 28 (16-40)  31 (18-44)  51 (28-74)***  30 (3-119)  39 (13-65)  27 (15-39) | 1  1.528 (0.948-2.465) | .777  **.077** |
| **GGT, U/l**  ≤ 100  > 100  **Bilirubin, mg/dl**  ≤ 1  > 1 | 74  50  107  19 | 18 (15-22)  10 (6-14)  14 (10-19)  42 (19-65)*** | 1  1.633 (1.05-2.541) | **.025**  .532 |  | 46 (7-85)  24 (14-34)  32 (23-41)  41 (11-71) | 1  1.906 (1.175-3.093) | **.007**  .892 |
| **Platelet count, 1/nl**  ≤ 250  > 250 | 61  65 | 14 (9-19)  12 (6-18) |  | .906 |  | 36 (20-52)  32 (18-46) |  | .860 |
| **INR**  ≤ 1  > 1 | 61  64 | 18 (13-23)  10 (6-14) |  | .166 |  | 61 (27-95)  31 (18-44) | 1  1.758 (1.074-2.878) | .**022** |
| **Hemoglobin, g/dl** |  |  |  | .225 |  |  |  | .154 |
| ≤ 12 | 26 | 12 (6-18) |  |  |  | 28 (12-44) |  |  |
| > 12 | 103 | 16 (11-21) |  |  |  | 36 (21-52) |  |  |
| **CRP, mg/l** |  |  |  | **.001** |  |  |  | **.001** |
| ≤ 10 | 70 | 18 (11-25) | 1 |  |  | 67 (55-80)*** | 1 |  |
| > 10 | 52 | 9 (6-13) | 2.098 (1.332-3.306) |  |  | 18 (13-23) | 2.645 (1.609-4.348) |  |
| **Operative time, min** |  |  |  | **.026** |  |  |  | .065 |
| ≤ 360 | 96 | 17 (12-22) | 1 |  |  | 38 (25-51) |  |  |
| > 360 | 31 | 8 (5-11) | 1.723 (1.050-2.825) |  |  | 24 (13-35) |  |  |
| **Operative procedure** |  |  |  | **.001** |  |  |  | **.028** |
| Monosegmentectomy + bisegmentectomy  Hemihepatectomy | 28  48 | 131 (96-167)***  17 (12-22) | 1  2.960 (1.354-6.468) |  |  | 140 (107-174)***  32 (19-45) | 1  2.231 (0.960-5.182) |  |
| Ext. Hemihepetectomy + Trisectionectomy | 43 | 9 (7-11) | 4.743 (2.179-10.324) |  |  | 29 (18-40) | 3.011 (1.318-6.879) |  |
| others | 11 | 8 (5-11) | 6.71 (2.66-16.925) |  |  | 27 (14-40) | 3.56 (1.324-9.569) |  |
| **In-hospital PRBC (qualitatively)** |  |  |  | .**024** |  |  |  | .**043** |
| No | 97 | 17 (11-23) | 1 |  |  | 38 (22-54) | 1 |  |
| Yes | 30 | 7 (5-9) | 1.717 (1.058-2.787) |  |  | 27 (7-47) | 1.662 (1.006-2.745) |  |
| **In-hospital FFP (qualitatively)** |  |  |  | **.016** |  |  |  | .**021** |
| No | 88 | 18 (13-23) | 1 |  |  | 39 (3-75) | 1 |  |
| Yes | 39 | 8 (5-11) | 1.723 (1.09-2.725) |  |  | 7 (11-39) | 1.740 (1.076-2.813) |  |
| **R1 resection** |  |  |  | **.001** |  |  |  | .**001** |
| No | 114 | 17 (12-22) | 1 |  |  | 38 (25-51) | 1 |  |
| Yes | 10 | 7 (6-8) | 3.21 (1.558-6.613) |  |  | 14 (5-23)*** | 4.400 (2.214-8.744) |  |
| **MVI** |  |  |  | **.005** |  |  |  | .**01** |
| No | 77 | 17 (10-24) | 1 |  |  | 46 (21-71) | 1 |  |
| Yes | 45 | 10 (6-14) | 1.841 (1.179-2.876) |  |  | 22 (16-28) | 1.847 (1.146-2.975) |  |
| **LVI** |  |  |  | .**001** |  |  |  | .**001** |
| No | 96 | 17 (12-22) | 1 |  |  | 41 (28-54) | 1 |  |
| Yes | 22 | 6 (3-9) | 2.512 (1.446-4.365) |  |  | 11 (3-19) | 3.337 (1.890-5.892) |  |
| **Tumor grading** |  |  |  | .105 |  |  |  | .**001** |
| G1 / G2 | 84 | 15 (9-21) |  |  |  | 46 (31-61) | 1 |  |
| G3 / G4 | 34 | 12 (7-17) |  |  |  | 16 (6-26) | 2.332 (1.397-3.894) |  |
| **pN category** |  |  |  | .**001** |  |  |  | .**001** |
| N0 | 78 | 19 (11-27) | 1 |  |  | 51 (27-75) | 1 |  |
| N1 | 41 | 7 (4-10) | 2.645 (1.641-4.263) |  |  | 13 (6-20) | 3.138 (1.899-5.183) |  |
| **ICU time, days** |  |  |  | .896 |  |  |  | .897 |
| ≤ 1 | 108 | 13 (8-18) |  |  |  | 32 (24-41) |  |  |
| > 1 | 21 | 15 (7-24) |  |  |  | 22 (9-35) |  |  |
| **Hospitalization, days** |  |  |  | **.007** |  |  |  | .112 |
| ≤ 14 | 74 | 18 (10-26) | 1 |  |  | 32 (23-41) |  |  |
| > 14 | 50 | 9 (6-12) | 1.806 (1.159-2.814) |  |  | 22 (12-32) |  |  |
| **Perioperative complications** |  |  |  | .125 |  |  |  | .**019** |
| Clavien-Dindo I/II/IIIa | 113 | 15 (11-19) |  |  |  | 38 (28-48) | 1 |  |
| Clavien-Dindo IIIb/IV | 14 | 8 (5-11) |  |  |  | 18 (5-31) | 2.116 (1.109-4.037) |  |
| **Adjuvant therapy** |  |  |  | .201 |  |  |  | .572 |
| No | 79 | 15 (8-22) |  |  |  | 38 (24-52) |  |  |
| Yes | 47 | 12 (7-17) |  |  |  | 29 (22-36) |  |  |

*Various parameters are associated with cancer-specific or recurrence -free survival. ASA, American society of anesthesiologists classification; AST, aspartate aminotransferase; BMI, body mass index; CSS, cancer-specific survival; FFP, fresh frozen plasma; pCCA, perihilar cholangiocarcinoma; GGT, gamma glutamyltransferase; iCCA; intrahepatic cholangiocarcinoma; INR, international normalized ratio; LVI, lympho-vascular invasion; MVI, microvascular invasion; PRBC, packed red blood cells; PVE, portal vein ligation. RFS, recurrence-free survival; UICC, Union for international cancer control.*

***Data presented as mean.

**Supplementary table 5:** Multivariable analysis of cancer-specific and recurrence-free survival in perihilar cholangiocarcinoma

|  |  | ***Recurrence-free survival (RFS)*** | | |  | ***Cancer-specific survival (CSS)*** | | |
| --- | --- | --- | --- | --- | --- | --- | --- | --- |
|  |  | **Relative risk (95% CI)** | | ***P* value** |  | **Relative risk (95% CI)** | | ***P* value** |
| **INR** |  |  |  | .128 |  |  |  | .074 |
| ≤ 1 |  |  |  |  |  |  |  |  |
| > 1 |  |  |  |  |  |  |  |  |
| **Hemoglobin, g/dl** |  |  |  | **.003** |  |  |  | **.013** |
| ≤ 13 |  | 1 |  |  |  | 1 |  |  |
| > 13 |  | 0.466 (0.283-0.767) |  |  |  | 0.506 (0.296-0.865) |  |  |
| **Operative time, min** |  |  |  | .255 |  |  |  | .747 |
| ≤ 360 |  |  |  |  |  |  |  |  |
| > 360 |  |  |  |  |  |  |  |  |
| **In-hospital PRBC (qualitatively)** |  |  |  | .922 |  |  |  | .79 |
| No |  |  |  |  |  |  |  |  |
| Yes |  |  |  |  |  |  |  |  |
| **In-hospital FFP (qualitatively)** |  |  |  | **.018** |  |  |  | **.001** |
| No |  | 1 |  |  |  | 1 |  |  |
| Yes |  | 1.839 (1.111-3.044) |  |  |  | 2.804 (1.542-5.099) |  |  |
| **R1 resection** |  |  |  | .780 |  |  |  | .865 |
| No |  |  |  |  |  |  |  |  |
| Yes |  |  |  |  |  |  |  |  |
| **MVI** |  |  |  | .209 |  |  |  | **.001** |
| No |  |  |  |  |  | 1 |  |  |
| Yes |  |  |  |  |  | 2.584 (1.477-4.521) |  |  |
| **LVI** |  |  |  | **.008** |  |  |  | .152 |
| No |  | 1 |  |  |  |  |  |  |
| Yes |  | 2.065 (1.206-3.537) |  |  |  |  |  |  |
| **Tumor grading** |  |  |  | .149 |  |  |  | .178 |
| G1 / G2 |  |  |  |  |  |  |  |  |
| G3 / G4 |  |  |  |  |  |  |  |  |
| **pN category** |  |  |  | **.001** |  |  |  | **.005** |
| N0 |  | 1 |  |  |  | 1 |  |  |
| N1 |  | 2.314 (1.379-3.883) |  |  |  | 2.132 (1.252-3.631) |  |  |
| **ICU time, days** |  |  |  | .185 |  |  |  | **.042** |
| ≤ 1 |  |  |  |  |  | 1 |  |  |
| > 1 |  |  |  |  |  | 1.755 (1.02-3.021) |  |  |
| **Hospitalization, days** |  |  |  | .563 |  |  |  | .669 |
| ≤ 14 |  |  |  |  |  |  |  |  |
| > 14 |  |  |  |  |  |  |  |  |
| **Adjuvant therapy** |  |  |  | .887 |  |  |  | .222 |
| No |  |  |  |  |  |  |  |  |
| Yes |  |  |  |  |  |  |  |  |

*Relative risks are only provided for significant parameters. CSS, cancer-specific survival; CI, confidence interval; FFP, fresh frozen plasma; pCCA, perihilar cholangiocarcinoma; iCCA; intrahepatic cholangiocarcinoma; LVI, lympho-vascular invasion; MVI, microvascular invasion; PRBC, packed red blood cells; PVE, portal vein ligation. RFS, recurrence-free survival; UICC, Union for international cancer control*

**Supplementary table 6:** Multivariable analysis of cancer-specific and recurrence-free survival in intrahepatic cholangiocarcinoma

|  |  | ***Recurrence-free survival (RFS)*** | | |  | ***Cancer-specific survival (CSS)*** | | |
| --- | --- | --- | --- | --- | --- | --- | --- | --- |
|  |  | **Relative risk (95% CI)** | | ***P* value** |  | **Relative risk (95% CI)** | | ***P* value** |
| **AST, U/l** |  |  |  |  |  |  |  | .092 |
| ≤ 40 |  |  |  |  |  |  |  |  |
| > 40 |  |  |  |  |  |  |  |  |
| **GGT, U/l** |  |  |  | .200 |  |  |  | .2 |
| ≤ 100 |  |  |  |  |  |  |  |  |
| > 100 |  |  |  |  |  |  |  |  |
| **INR** |  |  |  |  |  |  |  | .128 |
| ≤ 1 |  |  |  |  |  |  |  |  |
| > 1 |  |  |  |  |  |  |  |  |
| **CRP, mg/l**  ≤ 10  > 10 |  |  |  | .665 |  |  |  | .869 |
| **Operative time, min** |  |  |  | .212 |  |  |  |  |
| ≤ 360 |  |  |  |  |  |  |  |  |
| > 360 |  |  |  |  |  |  |  |  |
| **Operative procedure** |  |  |  | **. 019** |  |  |  | .642 |
| Monosegmentectomy + bisegmentectomy  Hemihepatectomy |  | 1  3.714 (1.240-11.123) |  |  |  |  |  |  |
| Ext. Hemihepatectomy + Trisectionectomy |  | 6.907 (2.305-20.697) |  |  |  |  |  |  |
| others |  | 12.751 (3.672-44.282) |  |  |  |  |  |  |
| **In-hospital PRBC (qualitatively)** |  |  |  | .816 |  |  |  | .549 |
| No |  |  |  |  |  |  |  |  |
| Yes |  |  |  |  |  |  |  |  |
| **In-hospital FFP (qualitatively)** |  |  |  | .897 |  |  |  | .408 |
| No |  |  |  |  |  |  |  |  |
| Yes |  |  |  |  |  |  |  |  |
| **R1 resection** |  |  |  | **. 039** |  |  |  | **.016** |
| No |  | 1 |  |  |  | 1 |  |  |
| Yes |  | 2.287 (1.043-5.013) |  |  |  | 2.746 (1.209-6.240) |  |  |
| **MVI** |  |  |  | .339 |  |  |  | .176 |
| No |  |  |  |  |  |  |  |  |
| Yes |  |  |  |  |  |  |  |  |
| **LVI** |  |  |  | .142 |  |  |  | .109 |
| No |  |  |  |  |  |  |  |  |
| Yes |  |  |  |  |  |  |  |  |
| **Tumor grading** |  |  |  |  |  |  |  | **0.023** |
| G1 / G2 |  |  |  |  |  | 1 |  |  |
| G3 / G4 |  |  |  |  |  | 2.033 (1.105-3.741) |  |  |
| **pN category** |  |  |  | **.001** |  |  |  | .**002** |
| N0 |  | 1 |  |  |  | 1 |  |  |
| N1 |  | 2.754 (1.552-4.887) |  |  |  | 2.521 (1.401-4.537) |  |  |
| **Hospitalization, days** |  |  |  | .594 |  |  |  |  |
| ≤ 14 |  |  |  |  |  |  |  |  |
| > 14 |  |  |  |  |  |  |  |  |
| **Perioperative complications** |  |  |  |  |  |  |  | 0.581 |
| Clavien-Dindo I/II/IIIa |  |  |  |  |  |  |  |  |
| Clavien-Dindo IIIb/IV |  |  |  |  |  |  |  |  |

*Relative risks are only provided for significant parameters. CSS, cancer-specific survival; CI, confidence interval; FFP, fresh frozen plasma; pCCA, perihilar cholangiocarcinoma; iCCA; intrahepatic cholangiocarcinoma; LVI, lympho-vascular invasion; MVI, microvascular invasion; PRBC, packed red blood cells; PVE, portal vein ligation. RFS, recurrence-free survival; UICC, Union for international cancer control*

**Supplementary table 7: Patients’ characteristics (Cholangiocarcinoma including perioperative mortality)**

| **Demographics** | **CCA (n=345)** | **Time to surgery subanalysis** | | | | **p value** |
| --- | --- | --- | --- | --- | --- | --- |
|  |  | **1 – 30 days**  **(n=108)** | **31 – 60 days**  **(n=135)** | **61 – 90 days**  **(n=48)** | **>90 days**  **(n=54)** |  |
| Gender, m/f (%) | 194 (56.2) / 151 (43.8) | 56 (51.9) / 52 (108) | 84 (62.2) / 51 (37.8) | 29 (60.4) / 19 (39.6) | 23 (46.3) / 29 (53.7) | .150 |
| Age (years) | 68 ± 11 | 63 ± 11 | 69 ± 10 | 68 _­_± 11 | 67 ± 12 | **.044** |
| BMI (kg/m^2^) | 25 ± 5 | 25 ± 5 | 26 ± 5 | 25 ± 4 | 26 ± 5 | .653 |
| Portal vein embolization, n (%) | 83 (24.2) | 9 (8.5) | 36 (26.7) | 20 (41.7) | 18 (33.3) | **.001** |
| ASA, n (%) |  |  |  |  |  | .227 |
| I | 12 (3.5) | 5 (4.7) | 6 (4.4) | 1 (2.1) | 0 |  |
| II | 125 (36.3) | 47 (43.9) | 42 (31.1) | 17 (35.4) | 19 (35.2) |  |
| III | 190 (55.2) | 48 (44.9) | 80 (59.3) | 30 (62.5) | 32 (59.3) |  |
| IV | 17 (4.9) | 7 (6.5) | 7 (5.2) | 0 | 3 (5.6) |  |
| V | 0 | 0 | 0 | 0 | 0 |  |
| Preoperative Chemotherapy | 0 | 0 | 0 | 0 | 0 | n.a. |
| Future liver remnant modulation |  |  |  |  |  |  |
| endoscopic stenting | 154 (44.9) | 46 (43.4) | 67 (49.6) | 21 (43.8) | 20 (37) |  |
| Percutaneous transhepatic biliary drainage | 50 (14.6) | 14 (13.2) | 23 (17) | 12 (25) | 1 (1.9) |  |
| Exploratory laparotomies during the same study period, n (%)** | n.a. | n.a. | n.a. | n.a. | n.a. | n.a. |
| **Clinical chemistry (preoperative)** |  |  |  |  |  |  |
| AST (U/l) | 39 ± 177 | 45 ± 279 | 39 ± 112 | 41 ± 115 | 31 ± 35 | .004 |
| GGT (U/l) | 242 ± 481 | 283 ± 549 | 246 ± 477 | 242 ± 446 | 118 ± 336 | .024 |
| Total bilirubin (mg/dl) | 0.6 ± 3.2 | 0.9 ± 4.7 | 0.8 ± 1.9 | 0.6 ± 2.7 | 0.4 ± 1.2 | **.001** |
| Hemoglobin (g/dl) | 12.5 ± 1.7 | 12.8 ± 1.7 | 12.5 ± 1.7 | 12.6 ± 2 | 12.2 ± 1.6 | .730 |
| Platelet count (/nl) | 276 ± 117 | 299 ± 121 | 262 ± 124 | 283 ± 96 | 255 ± 100 | **.004** |
| INR | 1 ± 5.26 | 1.01 ± 0.12 | 1.02 ± 0.11 | 1.04 ± 3.95 | 1.01 ± 12.8 | .555 |
| Prothrombin time (%) | 98 ± 16 | 98 ± 16 | 97 ± 15 | 96 ± 16 | 99 ± 16 | .678 |
| CRP (mg/l) | 11 ± 38 | 15 ± 34 | 10 ± 42 | 11 ± 50 | 10 ± 14 | .286 |
| **Operative Data** |  |  |  |  |  |  |
| Operative time (minutes) | 380 ± 128 | 366 ± 127 | 383 ± 125 | 400 ± 128 | 384 ± 140 | .619 |
| Operative procedure, n (%) |  |  |  |  |  | .198 |
| Atypical | 17 (5) | 3 (2.8) | 7 (5.2) | 4 (8.3) | 3 (5.6) |  |
| Monosegmentectomy | 1 (0.3) | 0 | 0 | 1 (2.1) | 0 |  |
| Bisegmentectomy | 14 (4.1) | 5 (4.7) | 6 (4.4) | 0 | 3 (5.6) |  |
| Hemihepatectomy | 102 (29.3) | 38 (35.8) | 39 (28.9) | 12 (25) | 13 (24.2) |  |
| Extended hemihepatectomy^#^ | 113 (32.9) | 36 (33.9) | 42 (31.1) | 16 (33.4) | 19 (35.2) |  |
| Trisectionectomy | 50 (14.7) | 11 (10.4) | 25 (18.4) | 7 (14.6) | 7 (13.1) |  |
| Hepatoduodenoectomy^†^ | 10 (2.9) | 2 (1.8) | 5 (3.7) | 2 (4.2) | 1 (1.9) |  |
| ALPPS | 16 (4.7) | 4 (3.8) | 4 (3) | 4 (8.3) | 4 (7.4) |  |
| other | 20 (5.8) | 7 (6.6) | 7 (5.2) | 2 (4.2) | 4 (7.5) |  |
| Laparoscopic resection, n (%) | 17 (6.2) | 4 (4.9) | 8 (7.1) | 2 (4.9) | 3 (7.5) | .887 |
| Intraoperative PRBC, n (%) | 142 (31.4) | 46 (43.4) | 50 (37) | 20 (41.7) | 26 (48.1) | .595 |
| Intraoperative FFP, n (%) | 160 (46.6) | 51 (48.1) | 62 (45.9) | 23 (47.9) | 24 (44.4) | .998 |
| Intraoperative platelets, n (%) | 5 (1.5) | 1 (0.9) | 1 (0.7) | 0 | 3 (5.6) | .052 |
| **Pathological examination** |  |  |  |  |  |  |
| R1 resection, n (%) | 51 (15) | 14 (13.3) | 17 (12.6) | 11 (22.9) | 9 (17.3) | .175 |
| pN category, n (%) |  |  |  |  |  | .117 |
| N0 | 191 (57.9) | 70 (66.7) | 73 (56.6) | 23 (48.9) | 25 (51) |  |
| N1 | 139 (42.1) | 35 (33.3) | 56 (43.4) | 24 (51.1) | 24 (49) |  |
| Tumor grading, n (%) |  |  |  |  |  | .757 |
| G1 | 7 (2.2) | 2 (1.9) | 3 (2.3) | 1 (2.1) | 1 (2.4) |  |
| G2 | 213 (66.6) | 72 (69.2) | 84 (65.6) | 31 (66) | 26 (63.4) |  |
| G3 | 92 (28.8) | 30 (28.8) | 37 (28.9) | 14 (29.8) | 11 (26.8) |  |
| G4 | 5 (1.9) | 0 | 3 (2.3) | 1 (2.1) | 2 (4.9) |  |
| MVI, n (%) | 105 (31.6) | 38 (36.5) | 43 (33.1) | 15 (31.3) | 9 (18) | .148 |
| LVI, n (%) | 77 (23.8) | 27 (27) | 26 (20.6) | 14 (29.2) | 10 (20) | .247 |
| pT category n (%) |  |  |  |  |  | .056 |
| Cis  1 | 1 (0.3)  72 (21.2) | 0  20 (18.9) | 1 (0.7)  22 (16.3) | 0  11 (22.9) | 0  19 (37.3) |  |
| 2 | 170 (49.3) | 50 (47.2) | 77 (57) | 24 (50) | 19 (37.2) |  |
| 3 | 66 (19.4) | 30 (28.3) | 21 (15.6) | 7 (14.6) | 8 (15.7) |  |
| 4 | 31 (9.1) | 6 (5.7) | 14 (10.4) | 6 (12.5) | 5 (9.8) |  |
| **Postoperative Data** |  |  |  |  |  |  |
| Intensive care, days | 1 ± 9 | 1 ± 3 | 1 ± 11 | 1 ± 13 | 1 ± 5 | .258 |
| Hospitalization, days | 15 ± 24 | 14 ± 18 | 15 ± 30 | 21 ± 23 | 22 ± 16 | .206 |
| Postoperative complications, n (%) |  |  |  |  |  | **.019** |
| No complications | 88 (25.7) | 31 (29.2) | 35 (25.9) | 5 (10.4) | 17 (31.5) |  |
| Clavien-Dindo I | 17 (5) | 5 (4.7) | 6 (4.4) | 2 (4.2) | 4 (7.4) |  |
| Clavien-Dindo II | 66 (19.2) | 22 (20.8) | 32 (23.7) | 8 (16.7) | 4 (7.4) |  |
| Clavien-Dindo IIIa | 66 (19.2) | 17 (16) | 25 (18.5) | 13 (27.1) | 11 (20.4) |  |
| Clavien-Dindo IIIb | 44 (12.8) | 14 (13.2) | 15 (11.1) | 5 (10.4) | 10 (18.5) |  |
| Clavien-Dindo IVa | 16 (4.7) | 6 (5.7) | 7 (5.2) | 2 (4.2) | 1 (1.9) |  |
| Clavien-Dindo IVb | 7 (2) | 0 | 3 (2.2) | 2 (4.2) | 2 (3.7) |  |
| Clavien-Dindo V | 39 (11.4) | 11 (10.4) | 12 (8.9) | 11 (22.9) | 5 (9.3) |  |
| **Oncologic Data*** |  |  |  |  |  |  |
| Adjuvant chemotherapy, n (%) | 102 (30) | 38 (35.8) | 37 (27.6) | 13 (28.3) | 14 (25.9) | .460 |
| Recurrence, n (%) | 179 (53.3) | 58 (55.8) | 73 (55.3) | 21 (45.7) | 27 (50) | .618 |
| Median RFS, months (95% CI) | 19 (13-24) | 17 (8-26) | 21 (15-27) | 28 (11-45) | 17 (9-25) | 838 |
| Median CSS, months (95% CI) | 29 (25-33) | 31 (25-37) | 31 (22-40) | 40 (28-53)*** | 21 (8-34) | .515 |
| Median OS, months (95% CI) | 25 (20-30) | 28 (22-34) | 26 (20-32) | 35 (24-47)*** | 16 (12-20) | .280 |

*Data presented as mean and standard deviation if not noted otherwise. *Data presented as median and interquartile range. #Right or left hepatectomy were considered to be extended hepatectomies if the middle hepatic vein was removed and the resection was extended into the segments IV or V/VIII, respectively. Procedures were defined as hepatoduodenoectomy if a major liver resections was combined with the concomitant resection of the pancreatic head. ALPPS, Associating liver partition and portal vein ligation for staged hepatectomy; ASA, American society of anesthesiologists classification; AST, aspartate aminotransferase; BMI, body mass index; CSS, cancer-specific survival; EBD, endoscopic biliary drainage; FFP, fresh frozen plasma; pCCA, perihilar cholangiocarcinoma; GGT, gamma glutamyltransferase; iCCA; intrahepatic cholangiocarcinoma; INR, international normalized ratio; LVI, lympho-vascular invasion; MVI, microvascular invasion;* *n./a., not applicable; OS, overall survival; PRBC, packed red blood cells; RFS, disease free survival; UICC, Union for international cancer control.* **This data was not included in statistical analysis. ***Data presented as mean.
